# Supplementary material for: A putative origin of the insect chemosensory receptor superfamily in the last common eukaryotic ancestor
Source: eLife. 2020 Dec 4;9:e62507. doi: 10.7554/eLife.62507 (PMC7746228; doi:10.7554/eLife.62507)
Supplement: Supplementary file 2. [file elife-62507-supp2.zip › 201130_SuppFile2_TOPCONS/query.result.html]

TOPCONS2 predictions


#### 

| No. | Length | numTM | SignalPeptide | RunTime(s) | SequenceName | Prediction | Source |
| --- | --- | --- | --- | --- | --- | --- | --- |
| 1 | 425 | 7 | No | 0.0 | SpunGRL1 gi|907093037|gb|KNC99049.1| hypothetical protein SPPG\_06000 [Spizellomyces punctatus DAOM BR117] | Fig\_all Fig\_topcons  Dumped prediction  deltaG  Topology view | cached |
| 2 | 426 | 7 | No | 0.0 | SpalGRL1 TPX68946.1 hypothetical protein SpCBS45565\_g02777 [Spizellomyces sp. 'palustris'] | Fig\_all Fig\_topcons  Dumped prediction  deltaG  Topology view | cached |
| 3 | 536 | 8 | No | 0.0 | TtraGRL1 gi|923135227|ref|XP\_013761079.1| hypothetical protein AMSG\_02047 [Thecamonas trainees ATCC 50062] >gi|906562792|gb|KNC56035.1| hypothetical protein AMSG\_02047 [Thecamonas trahens ATCC 50062] | Fig\_all Fig\_topcons  Dumped prediction  deltaG  Topology view | cached |
| 4 | 504 | 7 | No | 0.0 | TtraGRL2 gi|923120380|ref|XP\_013753662.1| hypothetical protein AMSG\_10508 [Thecamonas trahens ATCC 50062] >gi|906561266|gb|KNC54509.1| hypothetical protein AMSG\_10508 [Thecamonas trahens ATCC 50062] | Fig\_all Fig\_topcons  Dumped prediction  deltaG  Topology view | cached |
| 5 | 383 | 7 | No | 0.0 | TtraGRL4 XP\_013759396.1 hypothetical protein AMSG\_02477, partial [Thecamonas trahens ATCC 50062] | Fig\_all Fig\_topcons  Dumped prediction  deltaG  Topology view | cached |
| 6 | 340 | 8 | No | 0.0 | TtraGRL5 XP\_013757274.1 hypothetical protein AMSG\_05888 [Thecamonas trahens ATCC 50062] | Fig\_all Fig\_topcons  Dumped prediction  deltaG  Topology view | cached |
| 7 | 425 | 7 | No | 0.0 | TtraGRL6 XP\_013755387.1 hypothetical protein AMSG\_08306 [Thecamonas trahens ATCC 50062] | Fig\_all Fig\_topcons  Dumped prediction  deltaG  Topology view | cached |
| 8 | 410 | 2 | No | 0.0 | PfunGRL1 Protostelium aurantium var. fungivorum PRP89608.1 | Fig\_all Fig\_topcons  Dumped prediction  deltaG  Topology view | cached |
| 9 | 525 | 7 | No | 0.0 | VbraGRL1 gi|873229487|emb|CEM13019.1| unnamed protein product [Vitrella brassicaformis CCMP3155] | Fig\_all Fig\_topcons  Dumped prediction  deltaG  Topology view | cached |
| 10 | 500 | 7 | No | 0.0 | VbraGRL2 gi|873240772|emb|CEL93132.1| unnamed protein product [Vitrella brassicaformis CCMP3155] | Fig\_all Fig\_topcons  Dumped prediction  deltaG  Topology view | cached |
| 11 | 730 | 4 | No | 0.0 | VbraGRL3 CEM19221.1 unnamed protein product [Vitrella brassicaformis CCMP3155] | Fig\_all Fig\_topcons  Dumped prediction  deltaG  Topology view | cached |
| 12 | 670 | 7 | No | 0.0 | CpriGRL1 QDZ19318.1 hypothetical protein A3770\_02p18360 [Chloropicon primus] | Fig\_all Fig\_topcons  Dumped prediction  deltaG  Topology view | cached |
| 13 | 701 | 7 | No | 0.0 | MpusGRL1 Micromonas pusilla XP\_003054778.1 | Fig\_all Fig\_topcons  Dumped prediction  deltaG  Topology view | cached |
| 14 | 440 | 7 | No | 553.9 | TtraGRL3 gi|923132535|ref|XP\_013759733.1| hypothetical protein AMSG\_03829 [Thecamonas trahens ATCC 50062] gi|906554152|gb|KNC47395.1| hypothetical protein AMSG\_03829 [Thecamonas trahens ATCC 50062] CFIX | Fig\_all Fig\_topcons  Dumped prediction  deltaG  Topology view | newrun |
| 15 | 720 | 7 | No | 846.1 | VbraGRL4 CEM01650.1 unnamed protein product [Vitrella brassicaformis CCMP3155] | Fig\_all Fig\_topcons  Dumped prediction  deltaG  Topology view | newrun |
| 16 | 774 | 7 | No | 847.1 | VbraGRL5 CEM10760.1 unnamed protein product [Vitrella brassicaformis CCMP3155] | Fig\_all Fig\_topcons  Dumped prediction  deltaG  Topology view | newrun |
| 17 | 788 | 6 | No | 919.6 | VbraGRL6 CEM25255.1 unnamed protein product [Vitrella brassicaformis CCMP3155] | Fig\_all Fig\_topcons  Dumped prediction  deltaG  Topology view | newrun |
