# Supplementary material for: A putative origin of the insect chemosensory receptor superfamily in the last common eukaryotic ancestor
Source: eLife. 2020 Dec 4;9:e62507. doi: 10.7554/eLife.62507 (PMC7746228; doi:10.7554/eLife.62507)
Supplement: Supplementary file 2. [file elife-62507-supp2.zip › 201130_SuppFile2_TOPCONS/seq_9/nicetop.html]

|  |  |
| --- | --- |
|  | 1                                           41 |
| Seq. | MDGSSSTSTN EESDKNQRED AKADLATADT DARQKQGTEA TSSIRRLEHV |
| TOPCONS | iiiiiiiiii iiiiiiiiii iiiiiiiiii iiiiiiiiii iiiiiiiiii |
| OCTOPUS | iiiiiiiiii iiiiiiiiii iiiiiiiiii iiiiiiiiii iiiiiiiiii |
| Philius | iiiiiiiiii iiiiiiiiii iiiiiiiiii iiiiiiiiii iiiiiiiiii |
| PolyPhobius | iiiiiiiiii iiiiiiiiii iiiiiiiiii iiiiiiiiii iiiiiiiiii |
| SCAMPI | oooooooooo oooooooooo oooooooooo oooooooooo oooooooooo |
| SPOCTOPUS | iiiiiiiiii iiiiiiiiii iiiiiiiiii iiiiiiiiii iiiiiiiiii |
| PDB-homology |  |
|  | |
|  | 51                                          91 |
| Seq. | TTDLIEDEAL KWMVLLLRIT FLVPHTRAER VVAAVAMLVP WLSAVQQLLF |
| TOPCONS | iiiiiiiiii iiiiiiiiii iiiiiiiiiM MMMMMMMMMM MMMMMMMMMM |
| OCTOPUS | iiiiiiiiii iiiiiiiiii iiiiiiiiiM MMMMMMMMMM MMMMMMMMMM |
| Philius | iiiiiiiiii iiiiiiiiii iiiiiiiiii MMMMMMMMMM MMMMMMMMMM |
| PolyPhobius | iiiiiiiiii iiiiiiiiii iiiiiiiiii MMMMMMMMMM MMMMMMMMMM |
| SCAMPI | oooooooMMM MMMMMMMMMM MMMMMMMMii MMMMMMMMMM MMMMMMMMMM |
| SPOCTOPUS | iiiiiiiiii iiiiiiiiii iiiiiiiiiM MMMMMMMMMM MMMMMMMMMM |
| PDB-homology |  |
|  | |
|  | 101                                         141 |
| Seq. | LVDLDAVEAN DDRAVLLTNS ALWHVLTGCV RLWLLLCVPK QKLCKLFKCL |
| TOPCONS | oooooooooo oooooooMMM MMMMMMMMMM MMMMMMMMii iiiiiiiiii |
| OCTOPUS | oooooooooo oooooooMMM MMMMMMMMMM MMMMMMMMii iiiiiiiiii |
| Philius | oooooooooo oooooooooM MMMMMMMMMM MMMMMiiiii iiiiiiiiii |
| PolyPhobius | Mooooooooo oooooMMMMM MMMMMMMMMM MMMMMMMMii iiiiiiiiii |
| SCAMPI | Mooooooooo oooooMMMMM MMMMMMMMMM MMMMMMiiii iiiiiiiiii |
| SPOCTOPUS | oooooooooo oooooooMMM MMMMMMMMMM MMMMMMMMii iiiiiiiiii |
| PDB-homology |  |
|  | |
|  | 151                                         191 |
| Seq. | LRSSYWHMKP PPAVAFSTER LNRTAKIWSI VCFCTGVLNW ALLFFGYFIG |
| TOPCONS | iiiiiiiiii iiiiiiiiii iiiiiiiiMM MMMMMMMMMM MMMMMMMMMo |
| OCTOPUS | iiiiiiiiii iiiiiiiiii iiiiiiiiMM MMMMMMMMMM MMMMMMMMMo |
| Philius | iiiiiiiiii iiiiiiiiii iiiiiiMMMM MMMMMMMMMM MMMMMMMMMo |
| PolyPhobius | iiiiiiiiii iiiiiiiiii iiiiiiMMMM MMMMMMMMMM MMMMMMMMMM |
| SCAMPI | iiiiiiiiii iiiiiiiiii iiiiiiMMMM MMMMMMMMMM MMMMMMMooo |
| SPOCTOPUS | iiiiiiiiii iiiiiiiiii iiiiiiiiMM MMMMMMMMMM MMMMMMMMMo |
| PDB-homology |  |
|  | |
|  | 201                                         241 |
| Seq. | AEIRLVINYP YAQPDGQGWT IQEEEPGIYW WAVLQLVLQG LCSFAWIVPL |
| TOPCONS | oooooooooo oooooooooo oooooooooo oMMMMMMMMM MMMMMMMMMM |
| OCTOPUS | oooooooooo oooooooooo oooooooooo oMMMMMMMMM MMMMMMMMMM |
| Philius | oooooooooo oooooooooo oooooooooo oooooMMMMM MMMMMMMMMM |
| PolyPhobius | oooooooooo oooooooooo oooooooMMM MMMMMMMMMM MMMMMMMMMM |
| SCAMPI | oooooooooo oooooooooo oooooooooo ooMMMMMMMM MMMMMMMMMM |
| SPOCTOPUS | oooooooooo oooooooooo oooooooooo oMMMMMMMMM MMMMMMMMMM |
| PDB-homology |  |
|  | |
|  | 251                                         291 |
| Seq. | LPYSLAVGLL HERFRHFGMA LDHLIPALGN SNELLGVHRD TDSDGQPTGK |
| TOPCONS | MMiiiiiiii iiiiiiiiii iiiiiiiiii iiiiiiiiii iiiiiiiiii |
| OCTOPUS | MMiiiiiiii iiiiiiiiii iiiiiiiiii iiiiiiiiii iiiiiiiiii |
| Philius | MMMMMMMMMi iiiiiiiiii iiiiiiiiii iiiiiiiiii iiiiiiiiii |
| PolyPhobius | MMMMMMiiii iiiiiiiiii iiiiiiiiii iiiiiiiiii iiiiiiiiii |
| SCAMPI | MMMiiiiiii iiiiiiiiii iiiiiiiiii iiiiiiiiii iiiiiiiiii |
| SPOCTOPUS | MMiiiiiiii iiiiiiiiii iiiiiiiiii iiiiiiiiii iiiiiiiiii |
| PDB-homology |  |
|  | |
|  | 301                                         341 |
| Seq. | VPSLAQLTVA HRQLCQAVLV VDRIFRPFVA TWFSVNSALT IFLIYRIVFF |
| TOPCONS | iiiiiiiiii iiiiiiiiii iiiiiiiiiM MMMMMMMMMM MMMMMMMMMM |
| OCTOPUS | iiiiiiiiii iiiiiiiiii iiiiiiiiiM MMMMMMMMMM MMMMMMMMMM |
| Philius | iiiiiiiiii iiiiiiiiii iiiiiiiMMM MMMMMMMMMM MMMMMMMMMo |
| PolyPhobius | iiiiiiiiii iiiiiiiiii iiiiiiiMMM MMMMMMMMMM MMMMMMMMMo |
| SCAMPI | iiiiiiiiii iiiiiiiiii iiiiiiiiiM MMMMMMMMMM MMMMMMMMMM |
| SPOCTOPUS | iiiiiiiiii iiiiiiiiii iiiiiiiiiM MMMMMMMMMM MMMMMMMMMM |
| PDB-homology |  |
|  | |
|  | 351                                         391 |
| Seq. | REGASSTLMG SFFFWLLTGL LLQGIVGYKA AKIYAWHDHL LSKCLQIQLP |
| TOPCONS | oooooooMMM MMMMMMMMMM MMMMMMMMii iiiiiiiiii iiiiiiiiii |
| OCTOPUS | oooooooMMM MMMMMMMMMM MMMMMMMMii iiiiiiiiii iiiiiiiiii |
| Philius | ooooooMMMM MMMMMMMMMM MMMMMMMiii iiiiiiiiii iiiiiiiiii |
| PolyPhobius | ooooooooMM MMMMMMMMMM MMMMMMMMMi iiiiiiiiii iiiiiiiiii |
| SCAMPI | oooooooooo MMMMMMMMMM MMMMMMMMMM Miiiiiiiii iiiiiiiiii |
| SPOCTOPUS | oooooooMMM MMMMMMMMMM MMMMMMMMii iiiiiiiiii iiiiiiiiii |
| PDB-homology |  |
|  | |
|  | 401                                         441 |
| Seq. | PDQHTNAPPP TPPPLAAPLT SRQSSRKPLL LLRSHSLDTS IAPMCEEALA |
| TOPCONS | iiiiiiiiii iiiiiiiiii iiiiiiiiii iiiiiiiiii iiiiiiiiii |
| OCTOPUS | iiiiiiiiii iiiiiiiiii iiiiiiiiii iiiiiiiiii iiiiiiiiii |
| Philius | iiiiiiiiii iiiiiiiiii iiiiiiiiii iiiiiiiiii iiiiiiiiii |
| PolyPhobius | iiiiiiiiii iiiiiiiiii iiiiiiiiii iiiiiiiiii iiiiiiiiii |
| SCAMPI | iiiiiiiiii iiiiiiiiii iiiiiiiiii iiiiiiiiii iiiiiiiiii |
| SPOCTOPUS | iiiiiiiiii iiiiiiiiii iiiiiiiiii iiiiiiiiii iiiiiiiiii |
| PDB-homology |  |
|  | |
|  | 451                                         491 |
| Seq. | QSTHIVTRRE ETSESGVQRE LELLRFAHQV SNLRPGLTVG GAVLLNWQLI |
| TOPCONS | iiiiiiiiii iiiiiiiiii iiiiiiiiii iiiiiiiiii iiiiMMMMMM |
| OCTOPUS | iiiiiiiiii iiiiiiiiii iiiiiiiiii iiiiiMMMMM MMMMMMMMMM |
| Philius | iiiiiiiiii iiiiiiiiii iiiiiiiiii iiiiiiiiii iiMMMMMMMM |
| PolyPhobius | iiiiiiiiii iiiiiiiiii iiiiiiiiii iiiiiiiiii iiiMMMMMMM |
| SCAMPI | iiiiiiiiii iiiiiiiiii iiiiiiiiii iiiiiiiiii iiiiMMMMMM |
| SPOCTOPUS | iiiiiiiiii iiiiiiiiii iiiiiiiiii iiiiiiiiii iiiiiiMMMM |
| PDB-homology |  |
|  | |

|  |  |
| --- | --- |
|  | 501                   521 |
| Seq. | GTAASVVASV FVFLYETRKG NTETE |
| TOPCONS | MMMMMMMMMM MMMMMooooo ooooo |
| OCTOPUS | oMMMMMMMMM MMMMMMiiii iiiii |
| Philius | MMMMMMMMMM MMMMMooooo ooooo |
| PolyPhobius | MMMMMMMMMM MMMMMooooo ooooo |
| SCAMPI | MMMMMMMMMM MMMMMooooo ooooo |
| SPOCTOPUS | MMMMMMMMMM MMMMMMMooo ooooo |
| PDB-homology |  |
