# Supplementary material for: A putative origin of the insect chemosensory receptor superfamily in the last common eukaryotic ancestor
Source: eLife. 2020 Dec 4;9:e62507. doi: 10.7554/eLife.62507 (PMC7746228; doi:10.7554/eLife.62507)
Supplement: Supplementary file 2. [file elife-62507-supp2.zip › 201130_SuppFile2_TOPCONS/seq_10/nicetop.html]

|  |  |
| --- | --- |
|  | 1                                           41 |
| Seq. | MNGSSVSSSG EPSKIQPEED GSAANGVEIE KSAVGLPPSV TTGERLLGED |
| TOPCONS | iiiiiiiiii iiiiiiiiii iiiiiiiiii iiiiiiiiii iiiiiiiiii |
| OCTOPUS | iiiiiiiiii iiiiiiiiii iiiiiiiiii iiiiiiiiii iiiiiiiiii |
| Philius | iiiiiiiiii iiiiiiiiii iiiiiiiiii iiiiiiiiii iiiiiiiiii |
| PolyPhobius | oooooooooo oooooooooo oooooooooo oooooooooo oooooooooo |
| SCAMPI | oooooooooo oooooooooo oooooooooo oooooooooo oooooooooM |
| SPOCTOPUS | iiiiiiiiii iiiiiiiiii iiiiiiiiii iiiiiiiiii iiiiiiiiii |
| PDB-homology |  |
|  | |
|  | 51                                          91 |
| Seq. | ALKWMFVLLR ASLLVPYGRA ERVASGFACL VCWYLAIIDA LFLEDTEDFF |
| TOPCONS | iiiiiiiiii iiiiiiiiii iiMMMMMMMM MMMMMMMMMM MMMooooooo |
| OCTOPUS | iiiiiiiiii iiiiiiiiii iiMMMMMMMM MMMMMMMMMM MMMooooooo |
| Philius | iiiiiiiiii iiiiiiiiii iiMMMMMMMM MMMMMMMMMM MMMooooooo |
| PolyPhobius | MMMMMMMMMM MMMMMMMiii iiMMMMMMMM MMMMMMMMMM MMMooooooo |
| SCAMPI | MMMMMMMMMM MMMMMMMMMM iiMMMMMMMM MMMMMMMMMM MMMooooooo |
| SPOCTOPUS | iiiiiiiiii iiiiiiiiii iiMMMMMMMM MMMMMMMMMM MMMooooooo |
| PDB-homology |  |
|  | |
|  | 101                                         141 |
| Seq. | TDTKGRGILL SATTLWFLMC GGIRLWMLVS VSRERLAMVF ACLVKGSYWT |
| TOPCONS | ooooooooMM MMMMMMMMMM MMMMMMMMMi iiiiiiiiii iiiiiiiiii |
| OCTOPUS | oooooooooo MMMMMMMMMM MMMMMMMMMM Miiiiiiiii iiiiiiiiii |
| Philius | oooooooMMM MMMMMMMMMM MMMMMMMMii iiiiiiiiii iiiiiiiiii |
| PolyPhobius | oooooooMMM MMMMMMMMMM MMMMMMMMMM iiiiiiiiii iiiiiiiiii |
| SCAMPI | ooooooooMM MMMMMMMMMM MMMMMMMMMi iiiiiiiiii iiiiiiiiii |
| SPOCTOPUS | oooooooooo MMMMMMMMMM MMMMMMMMMM Miiiiiiiii iiiiiiiiii |
| PDB-homology |  |
|  | |
|  | 151                                         191 |
| Seq. | LALGDQSKDV AESFSRRVKV WSVVCFAAAL TSWLLLVFGY IIGAQTRRVF |
| TOPCONS | iiiiiiiiii iiiiiiiiii iMMMMMMMMM MMMMMMMMMM MMoooooooo |
| OCTOPUS | iiiiiiiiii iiiiiiiiii iMMMMMMMMM MMMMMMMMMM MMoooooooo |
| Philius | iiiiiiiiii iiiiiiiiii MMMMMMMMMM MMMMMMMMMM MMMMoooooo |
| PolyPhobius | iiiiiiiiii iiiiiiiiiM MMMMMMMMMM MMMMMMMMMM MMMooooooo |
| SCAMPI | iiiiiiiiii iiiiiiiiiM MMMMMMMMMM MMMMMMMMMM oooooooooo |
| SPOCTOPUS | iiiiiiiiii iiiiiiiiii iMMMMMMMMM MMMMMMMMMM MMoooooooo |
| PDB-homology |  |
|  | |
|  | 201                                         241 |
| Seq. | NFPWADWFIE EESPGIHWFG LVTAILSIFP CFVWIVPLLP YSLAINLLHE |
| TOPCONS | oooooooooo oooooooooM MMMMMMMMMM MMMMMMMMMM iiiiiiiiii |
| OCTOPUS | oooooooooo oooooooooM MMMMMMMMMM MMMMMMMMMM iiiiiiiiii |
| Philius | oooooooooo oooooMMMMM MMMMMMMMMM MMMMMMMMii iiiiiiiiii |
| PolyPhobius | oooooooooo oooooMMMMM MMMMMMMMMM MMMMMMMMMM MMMiiiiiii |
| SCAMPI | oooooooooo oooooooooo MMMMMMMMMM MMMMMMMMMM Miiiiiiiii |
| SPOCTOPUS | oooooooooo oooooooooM MMMMMMMMMM MMMMMMMMMM iiiiiiiiii |
| PDB-homology |  |
|  | |
|  | 251                                         291 |
| Seq. | RFRAFCQSLD DSIRPLERQK TRPGEPSSYA EAAAGKKPSV RQLTLAHRQL |
| TOPCONS | iiiiiiiiii iiiiiiiiii iiiiiiiiii iiiiiiiiii iiiiiiiiii |
| OCTOPUS | iiiiiiiiii iiiiiiiiii iiiiiiiiii iiiiiiiiii iiiiiiiiii |
| Philius | iiiiiiiiii iiiiiiiiii iiiiiiiiii iiiiiiiiii iiiiiiiiii |
| PolyPhobius | iiiiiiiiii iiiiiiiiii iiiiiiiiii iiiiiiiiii iiiiiiiiii |
| SCAMPI | iiiiiiiiii iiiiiiiiii iiiiiiiiii iiiiiiiiii iiiiiiiiii |
| SPOCTOPUS | iiiiiiiiii iiiiiiiiii iiiiiiiiii iiiiiiiiii iiiiiiiiii |
| PDB-homology |  |
|  | |
|  | 301                                         341 |
| Seq. | CQGVMMVDRT FRAFVAISFT INTALSILVV YRIVFFGYTA SSAEAGAFVF |
| TOPCONS | iiiiiiiiii iiiiiMMMMM MMMMMMMMMM MMMMMMoooo ooMMMMMMMM |
| OCTOPUS | iiiiiiiiii iiiiiMMMMM MMMMMMMMMM MMMMMMoooo ooMMMMMMMM |
| Philius | iiiiiiiiii iiMMMMMMMM MMMMMMMMMM MMMMMooooo ooMMMMMMMM |
| PolyPhobius | iiiiiiiiii iiiMMMMMMM MMMMMMMMMM MMMMMooooo ooooMMMMMM |
| SCAMPI | iiiiiiiiii iiiiiMMMMM MMMMMMMMMM MMMMMMoooo oooooMMMMM |
| SPOCTOPUS | iiiiiiiiii iiiiiMMMMM MMMMMMMMMM MMMMMMoooo ooMMMMMMMM |
| PDB-homology |  |
|  | |
|  | 351                                         391 |
| Seq. | WCVGLAIQFF VCYKSAKLFS WHEHLRLRCH QVQVGLPYHA SSTPTSLASQ |
| TOPCONS | MMMMMMMMMM MMMiiiiiii iiiiiiiiii iiiiiiiiii iiiiiiiiii |
| OCTOPUS | MMMMMMMMMM MMMiiiiiii iiiiiiiiii iiiiiiiiii iiiiiiiiii |
| Philius | MMMMMMMMMM MMMiiiiiii iiiiiiiiii iiiiiiiiii iiiiiiiiii |
| PolyPhobius | MMMMMMMMMM MMMMiiiiii iiiiiiiiii iiiiiiiiii iiiiiiiiii |
| SCAMPI | MMMMMMMMMM MMMMMMiiii iiiiiiiiii iiiiiiiiii iiiiiiiiii |
| SPOCTOPUS | MMMMMMMMMM MMMiiiiiii iiiiiiiiii iiiiiiiiii iiiiiiiiii |
| PDB-homology |  |
|  | |
|  | 401                                         441 |
| Seq. | MTRCHSVADD LTSAGGPGGD SSTSSRRAVT QEALYARQER LELELEHLRF |
| TOPCONS | iiiiiiiiii iiiiiiiiii iiiiiiiiii iiiiiiiiii iiiiiiiiii |
| OCTOPUS | iiiiiiiiii iiiiiiiiii iiiiiiiiii iiiiiiiiii iiiiiiiiii |
| Philius | iiiiiiiiii iiiiiiiiii iiiiiiiiii iiiiiiiiii iiiiiiiiii |
| PolyPhobius | iiiiiiiiii iiiiiiiiii iiiiiiiiii iiiiiiiiii iiiiiiiiii |
| SCAMPI | iiiiiiiiii iiiiiiiiii iiiiiiiiii iiiiiiiiii iiiiiiiiii |
| SPOCTOPUS | iiiiiiiiii iiiiiiiiii iiiiiiiiii iiiiiiiiii iiiiiiiiii |
| PDB-homology |  |
|  | |
|  | 451                                         491 |
| Seq. | SHQVESFTPG ITVGGAVTMN WRVLGVAASV TASVFVFLYE TRKNNRGEGE |
| TOPCONS | iiiiiiiiii iiiiiiiiMM MMMMMMMMMM MMMMMMMMMo oooooooooo |
| OCTOPUS | iiiiiiiiii iiiiiiiiii MMMMMMMMMM MMMMMMMMMM Mooooooooo |
| Philius | iiiiiiiiii iiiiiiiiii MMMMMMMMMM MMMMMMMMMo oooooooooo |
| PolyPhobius | iiiiiiiiii iiiiiiiiMM MMMMMMMMMM MMMMMMMMMo oooooooooo |
| SCAMPI | iiiiiiiiii iiiiiiiiMM MMMMMMMMMM MMMMMMMMMo oooooooooo |
| SPOCTOPUS | iiiiiiiiii iiiiiiiiii MMMMMMMMMM MMMMMMMMMM Mooooooooo |
| PDB-homology |  |
|  | |
