# Supplementary material for: A putative origin of the insect chemosensory receptor superfamily in the last common eukaryotic ancestor
Source: eLife. 2020 Dec 4;9:e62507. doi: 10.7554/eLife.62507 (PMC7746228; doi:10.7554/eLife.62507)
Supplement: Supplementary file 2. [file elife-62507-supp2.zip › 201130_SuppFile2_TOPCONS/seq_11/nicetop.html]

|  |  |
| --- | --- |
|  | 1                                           41 |
| Seq. | MEGRSGSDPP ASSSAKVIEV PTVSSCATKV TTSLDLALDS RHDHSTLPVI |
| TOPCONS | oooooooooo oooooooooo oooooooooo oooooooooo oooooooooo |
| OCTOPUS |  |
| Philius | iiiiiiiiii iiiiiiiiii iiiiiiiiii iiiiiiiiii iiiiiiiiii |
| PolyPhobius | iiiiiiiiii iiiiiiiiii iiiiiiiiii iiiiiiiiii iiiiiiiiii |
| SCAMPI |  |
| SPOCTOPUS | oooooooooo oooooooooo oooooooooo oooooooooo oooooooooo |
| PDB-homology |  |
|  | |
|  | 51                                          91 |
| Seq. | MEEETLSRRL RKCCKCVHES LQILCCIPSS IIWKRRKMGV SMQKMDFVKR |
| TOPCONS | oooooooooo oooooooooo oooooooooo oooooooooo oooooooooo |
| OCTOPUS |  |
| Philius | iiiiiiiiii iiiiiiiiii iiiiiiiiii iiiiiiiiii iiiiiiiiii |
| PolyPhobius | iiiiiiiiii iiiiiiiiii iiiiiiiiii iiiiiiiiii iiiiiiiiii |
| SCAMPI |  |
| SPOCTOPUS | oooooooooo oooooooooo oooooooooo oooooooooo oooooooooo |
| PDB-homology |  |
|  | |
|  | 101                                         141 |
| Seq. | ELRTFVIWSR LVGLSFLPDD NNFVLWVWPV IIQVLLWHTT FVNTMGLWWS |
| TOPCONS | oooooooooo oooooooooo oooooooooo oooooooooo oooooooooo |
| OCTOPUS |  |
| Philius | iiiiiiiiii iiiiiiiiii iiiMMMMMMM MMMMMMMMMM MMoooooooo |
| PolyPhobius | iiiiiiiiii iiiiiiiiii iiMMMMMMMM MMMMMMMMMM MMMMMMMMMM |
| SCAMPI |  |
| SPOCTOPUS | oooooooooo oooooooooo oooMMMMMMM MMMMMMMMMM MMMMoooooo |
| PDB-homology |  |
|  | |
|  | 151                                         191 |
| Seq. | VTYQHQLQHM QMEASEANGS DAQQQQQQER GAAEMRQEEP LDPFVVFLFF |
| TOPCONS | oooooooooo oooooooooo oooooooooo oooooooooo oooMMMMMMM |
| OCTOPUS |  |
| Philius | oooooooooo oooooooooo oooooooooo oooooooooo oooMMMMMMM |
| PolyPhobius | oooooooooo oooooooooo oooooooooo oooooooooo oooMMMMMMM |
| SCAMPI |  |
| SPOCTOPUS | oooooooooo oooooooooo oooooooooo oooooooooo ooMMMMMMMM |
| PDB-homology |  |
|  | |
|  | 201                                         241 |
| Seq. | SASLAITVSY TVLRIQWYKK GATFGQVVEF HRARGGRFLR LLRGPIVARL |
| TOPCONS | MMMMMMMMMM MMMMiiiiii iiiiiiiiii iiiiiiiiii iiiMMMMMMM |
| OCTOPUS |  |
| Philius | MMMMMMMMMM MMMiiiiiii iiiiiiiiii iiiiiiiiii iiiiiMMMMM |
| PolyPhobius | MMMMMMMMMM MMMiiiiiii iiiiiiiiii iiiiiiiiii iiiiiMMMMM |
| SCAMPI |  |
| SPOCTOPUS | MMMMMMMMMM MMMooooooo oooooooooo oooooooooo oooMMMMMMM |
| PDB-homology |  |
|  | |
|  | 251                                         291 |
| Seq. | VISIVICIAS WLHFTLHEYI SKWVRGKAVG QYIMPGSEWE GLNVFVHVNV |
| TOPCONS | MMMMMMMMMM MMMMoooooo oooooooooo oooooooooo oooooooooo |
| OCTOPUS |  |
| Philius | MMMMMMMMMM MMMMoooooo oooooooooo oooooooooo oooooooooo |
| PolyPhobius | MMMMMMMMMM MMMMMMoooo oooooooooo oooooooooo oooMMMMMMM |
| SCAMPI |  |
| SPOCTOPUS | MMMMMMMMMM MMMMoooooo oooooooooo oooooooooo oooooooMMM |
| PDB-homology |  |
|  | |
|  | 301                                         341 |
| Seq. | TISAFLGYLV QPMIAVSVAG LLSTICQMHT AAVNCLLWEL SEPVRIQERQ |
| TOPCONS | oooooooooo oooooooooo oooooooooo oooooooooo oooooooooo |
| OCTOPUS |  |
| Philius | oooMMMMMMM MMMMMMMMMM MMMMMMiiii iiiiiiiiii iiiiiiiiii |
| PolyPhobius | MMMMMMMMMM MMMMMMMMMM MMMMMMMiii iiiiiiiiii iiiiiiiiii |
| SCAMPI |  |
| SPOCTOPUS | MMMMMMMMMM MMMMMMMMMM MMMMMMMMoo oooooooooo oooooooooo |
| PDB-homology |  |
|  | |
|  | 351                                         391 |
| Seq. | RKRRINKKLS AGQISRYDSS EQLPLMNAVT GHSTSGDHLA MDDDLRALSS |
| TOPCONS | oooooooooo oooooooooo oooooooooo oooooooooo oooooooooo |
| OCTOPUS |  |
| Philius | iiiiiiiiii iiiiiiiiii iiiiiiiiii iiiiiiiiii iiiiiiiiii |
| PolyPhobius | iiiiiiiiii iiiiiiiiii iiiiiiiiii iiiiiiiiii iiiiiiiiii |
| SCAMPI |  |
| SPOCTOPUS | oooooooooo oooooooooo oooooooooo oooooooooo oooooooooo |
| PDB-homology |  |
|  | |
|  | 401                                         441 |
| Seq. | LVQGGGEGRE GAGEDEEEAG VTYTPQRVAY LMDLHRKIDS LITRSSRAVQ |
| TOPCONS | oooooooooo oooooooooo oooooooooo oooooooooo oooooooooo |
| OCTOPUS |  |
| Philius | iiiiiiiiii iiiiiiiiii iiiiiiiiii iiiiiiiiii iiiiiiiiii |
| PolyPhobius | iiiiiiiiii iiiiiiiiii iiiiiiiiii iiiiiiiiii iiiiiiiiii |
| SCAMPI |  |
| SPOCTOPUS | oooooooooo oooooooooo oooooooooo oooooooooo oooooooooo |
| PDB-homology |  |
|  | |
|  | 451                                         491 |
| Seq. | LPIATMSLLY FITFLTCAFL FVFLPKKTQD VNMVICVIGV FITVGISCYW |
| TOPCONS | oooMMMMMMM MMMMMMMMMM MMMMiiiiii MMMMMMMMMM MMMMMMMMMM |
| OCTOPUS |  |
| Philius | iiMMMMMMMM MMMMMMMMMM MMMMoooooo MMMMMMMMMM MMMMMMMMMM |
| PolyPhobius | iMMMMMMMMM MMMMMMMMMM MMMMMooooo MMMMMMMMMM MMMMMMMMMM |
| SCAMPI |  |
| SPOCTOPUS | MMMMMMMMMM MMMMMMMMMM Mooooooooo ooMMMMMMMM MMMMMMMMMM |
| PDB-homology |  |
|  | |
|  | 501                                         541 |
| Seq. | LLRASSQVTA KCRRLGETAS LNPCPNALLH IDVDETALAD GEVEGFIHPQ |
| TOPCONS | Mooooooooo oooooooooo oooooooooo oooooooooo oooooooooo |
| OCTOPUS |  |
| Philius | MMMiiiiiii iiiiiiiiii iiiiiiiiii iiiiiiiiii iiiiiiiiii |
| PolyPhobius | MMiiiiiiii iiiiiiiiii iiiiiiiiii iiiiiiiiii iiiiiiiiii |
| SCAMPI |  |
| SPOCTOPUS | MMMooooooo oooooooooo oooooooooo oooooooooo oooooooooo |
| PDB-homology |  |
|  | |
|  | 551                                         591 |
| Seq. | TETPAYRASI LRDHTIVISA TNELAALPPL FPPSSPSTSP SHRDKNEPSP |
| TOPCONS | oooooooooo oooooooooo oooooooooo oooooooooo oooooooooo |
| OCTOPUS |  |
| Philius | iiiiiiiiii iiiiiiiiii iiiiiiiiii iiiiiiiiii iiiiiiiiii |
| PolyPhobius | iiiiiiiiii iiiiiiiiii iiiiiiiiii iiiiiiiiii iiiiiiiiii |
| SCAMPI |  |
| SPOCTOPUS | oooooooooo oooooooooo oooooooooo oooooooooo oooooooooo |
| PDB-homology |  |
|  | |
|  | 601                                         641 |
| Seq. | VVESPLSLTP PKQSRSLSEG EAVSNGSPTT APSAAASSSG GPPCVIGKQD |
| TOPCONS | oooooooooo oooooooooo oooooooooo oooooooooo oooooooooo |
| OCTOPUS |  |
| Philius | iiiiiiiiii iiiiiiiiii iiiiiiiiii iiiiiiiiii iiiiiiiiii |
| PolyPhobius | iiiiiiiiii iiiiiiiiii iiiiiiiiii iiiiiiiiii iiiiiiiiii |
| SCAMPI |  |
| SPOCTOPUS | oooooooooo oooooooooo oooooooooo oooooooooo oooooooooo |
| PDB-homology |  |
|  | |
|  | 651                                         691 |
| Seq. | AEVQTSGRDR AEDDASVRRP PCEDWLQQVM LVQFFSSTSS GWRVYDSLIT |
| TOPCONS | oooooooooo oooooooooo oooooooooo oooooooooo oooooooooo |
| OCTOPUS |  |
| Philius | iiiiiiiiii iiiiiiiiii iiiiiiiiii iiiiiiiiii iiiiiiiiii |
| PolyPhobius | iiiiiiiiii iiiiiiiiii iiiiiiiiii iiiiiiiiii iiiiiiiiii |
| SCAMPI |  |
| SPOCTOPUS | oooooooooo oooooooooo oooooooooo oooooooooo oooooooooo |
| PDB-homology |  |
|  | |

|  |  |
| --- | --- |
|  | 701                   721 |
| Seq. | PELLSRVVYG SVTIFAIAIQ RVVLSYGEVV |
| TOPCONS | oooooooooo oooooooooo oooooooooo |
| OCTOPUS |  |
| Philius | iiiiiiMMMM MMMMMMMMMM MMMMoooooo |
| PolyPhobius | iiiiiiMMMM MMMMMMMMMM MMMMMMoooo |
| SCAMPI |  |
| SPOCTOPUS | oooooooooo oooooooooo oooooooooo |
| PDB-homology |  |
