# Supplementary material for: A putative origin of the insect chemosensory receptor superfamily in the last common eukaryotic ancestor
Source: eLife. 2020 Dec 4;9:e62507. doi: 10.7554/eLife.62507 (PMC7746228; doi:10.7554/eLife.62507)
Supplement: Supplementary file 2. [file elife-62507-supp2.zip › 201130_SuppFile2_TOPCONS/seq_16/nicetop.html]

|  |  |
| --- | --- |
|  | 1                                           41 |
| Seq. | MARRIDSVPN IAETLGESSM SRVLPTHPIA RRRTATGAED GRASAGAEAS |
| TOPCONS | iiiiiiiiii iiiiiiiiii iiiiiiiiii iiiiiiiiii iiiiiiiiii |
| OCTOPUS | iiiiiiiiii iiiiiiiiii iiiiiiiiii iiiiiiiiii iiiiiiiiii |
| Philius | iiiiiiiiii iiiiiiiiii iiiiiiiiii iiiiiiiiii iiiiiiiiii |
| PolyPhobius | iiiiiiiiii iiiiiiiiii iiiiiiiiii iiiiiiiiii iiiiiiiiii |
| SCAMPI | iiiiiiiiii iiiiiiiiii iiiiiiiiii iiiiiiiiii iiiiiiiiii |
| SPOCTOPUS | iiiiiiiiii iiiiiiiiii iiiiiiiiii iiiiiiiiii iiiiiiiiii |
| PDB-homology |  |
|  | |
|  | 51                                          91 |
| Seq. | EEPPSSSGVR RTRTEPVDDA DGDGVGGGGG DDGGGGDDGG GGGGDDDDYP |
| TOPCONS | iiiiiiiiii iiiiiiiiii iiiiiiiiii iiiiiiiiii iiiiiiiiii |
| OCTOPUS | iiiiiiiiii iiiiiiiiii iiiiiiiiii iiiiiiiiii iiiiiiiiii |
| Philius | iiiiiiiiii iiiiiiiiii iiiiiiiiii iiiiiiiiii iiiiiiiiii |
| PolyPhobius | iiiiiiiiii iiiiiiiiii iiiiiiiiii iiiiiiiiii iiiiiiiiii |
| SCAMPI | iiiiiiiiii iiiiiiiiii iiiiiiiiii iiiiiiiiii iiiiiiiiii |
| SPOCTOPUS | iiiiiiiiii iiiiiiiiii iiiiiiiiii iiiiiiiiii iiiiiiiiii |
| PDB-homology |  |
|  | |
|  | 101                                         141 |
| Seq. | FSSPPSPSSA RASEDERGVV DELPALRSFA RLLRVVCVVP SRLPLSERDA |
| TOPCONS | iiiiiiiiii iiiiiiiiii iiiiiiiiii iiiiiiiiii iiiiiiiiii |
| OCTOPUS | iiiiiiiiii iiiiiiiiii iiiiiiiiii iiiiiiiiii iiiiiiiiii |
| Philius | iiiiiiiiii iiiiiiiiii iiiiiiiiii iiiiiiiiii iiiiiiiiii |
| PolyPhobius | iiiiiiiiii iiiiiiiiii iiiiiiiiii iiiiiiiiii iiiiiiiiii |
| SCAMPI | iiiiiiiiii iiiiiiiiii iiiiiiiiii iiiiiiiiii iiiiiiiiii |
| SPOCTOPUS | iiiiiiiiii iiiiiiiiii iiiiiiiiii iiiiiiiiii iiiiiiiiii |
| PDB-homology |  |
|  | |
|  | 151                                         191 |
| Seq. | DAAAAFAASA RRARATEAEG IVELAPLLST ATPPGASPRR RRRRRAARAL |
| TOPCONS | iiiiiiiiii iiiiiiiiii iiiiiiiiii iiiiiiiiii iiiiiiiiMM |
| OCTOPUS | iiiiiiiiii iiiiiiiiii iiiiiiiiii iiiiiiiiii iiiiiiMMMM |
| Philius | iiiiiiiiii iiiiiiiiii iiiiiiiiii iiiiiiiiii iiiiiiiiMM |
| PolyPhobius | iiiiiiiiii iiiiiiiiii iiiiiiiiii iiiiiiiiii iiiiiiiiMM |
| SCAMPI | iiiiiiiiii iiiiiiiiii iiiiiiiiii iiiiiiiiii iiiiiiiiii |
| SPOCTOPUS | iiiiiiiiii iiiiiiiiii iiiiiiiiii iiiiiiiiii iiiiiiMMMM |
| PDB-homology |  |
|  | |
|  | 201                                         241 |
| Seq. | ADVFCVLATM LLGLVAPLYR ARYEVVNAPD AAVTRLDAKA FAFLCMHFYV |
| TOPCONS | MMMMMMMMMM MMMMMMMMMo oooooooooo ooooooMMMM MMMMMMMMMM |
| OCTOPUS | MMMMMMMMMM MMMMMMMooo oooooooooo ooooooMMMM MMMMMMMMMM |
| Philius | MMMMMMMMMM MMMMMMMMMo oooooooooo oooooooMMM MMMMMMMMMM |
| PolyPhobius | MMMMMMMMMM MMMMMMMMMo oooooooooo ooooooooMM MMMMMMMMMM |
| SCAMPI | iiiiiiiiii iiiiiiiiii iiiiiiiiii iiiiiiiiii iiiiiiiiii |
| SPOCTOPUS | MMMMMMMMMM MMMMMMMooo oooooooooo ooooooMMMM MMMMMMMMMM |
| PDB-homology |  |
|  | |
|  | 251                                         291 |
| Seq. | CWYSLQVRYC ARFSLADVLF MRAAGSGACP ARHRKRYDDA PSTRFATPSP |
| TOPCONS | MMMMMMMiii iiiiiiiiii iiiiiiiiii iiiiiiiiii iiiiiiiiii |
| OCTOPUS | MMMMMMMiii iiiiiiiiii iiiiiiiiii iiiiiiiiii iiiiiiiiii |
| Philius | MMMMMMiiii iiiiiiiiii iiiiiiiiii iiiiiiiiii iiiiiiiiii |
| PolyPhobius | MMMMMiiiii iiiiiiiiii iiiiiiiiii iiiiiiiiii iiiiiiiiii |
| SCAMPI | iiiiiiiiii iiiiiiiiii iiiiiiiiii iiiiiiiiii iiiiiiiiii |
| SPOCTOPUS | MMMMMMMiii iiiiiiiiii iiiiiiiiii iiiiiiiiii iiiiiiiiii |
| PDB-homology |  |
|  | |
|  | 301                                         341 |
| Seq. | RHRHVISTVH DAYVGIVKRQ SWRELLRLLL PFVVLKLAAT AYVHVGGGAR |
| TOPCONS | iiiiiiiiii iiiiiiiiii iiiiMMMMMM MMMMMMMMMM MMMMMooooo |
| OCTOPUS | iiiiiiiiii iiiiiiiiii iiiiMMMMMM MMMMMMMMMM MMMMMooooo |
| Philius | iiiiiiiiii iiiiiiiiii iiiiiiiMMM MMMMMMMMMM MMMMMMoooo |
| PolyPhobius | iiiiiiiiii iiiiiiiiii iiiiiMMMMM MMMMMMMMMM MMMMMooooo |
| SCAMPI | iiiiiiiiii iiiiiiiiii iiiiiiiiii iiiiiiiiii iiiiiiiiii |
| SPOCTOPUS | iiiiiiiiii iiiiiiiiii iiiiMMMMMM MMMMMMMMMM MMMMMooooo |
| PDB-homology |  |
|  | |
|  | 351                                         391 |
| Seq. | KETVSYAIPG WWRRRRRGGG DAASLTGVGK AVAVSVGFVS ESYMAAVFLT |
| TOPCONS | oooooooooo oooooooooo oooooooooo oooooooooo MMMMMMMMMM |
| OCTOPUS | oooooooooo oooooooooo oooooooooo oooooooooo MMMMMMMMMM |
| Philius | oooooooooo oooooooooo oooooooooo oooooooooo oooMMMMMMM |
| PolyPhobius | oooooooooo oooooooooo oooooooooo oooooooooo ooMMMMMMMM |
| SCAMPI | iiiiiiiiii iiiiiiiiii iiiMMMMMMM MMMMMMMMMM MMMMoMMMMM |
| SPOCTOPUS | oooooooooo oooooooooo oooooooooo oooooooooo MMMMMMMMMM |
| PDB-homology |  |
|  | |
|  | 401                                         441 |
| Seq. | TCVLFRLTCA LVLLKLNAFL VMMRGTDDDD DEAAAAPAAT DDDDDDDDDD |
| TOPCONS | MMMMMMMMMM Miiiiiiiii iiiiiiiiii iiiiiiiiii iiiiiiiiii |
| OCTOPUS | MMMMMMMMMM Miiiiiiiii iiiiiiiiii iiiiiiiiii iiiiiiiiii |
| Philius | MMMMMMMMMM MMMMiiiiii iiiiiiiiii iiiiiiiiii iiiiiiiiii |
| PolyPhobius | MMMMMMMMMM MMMMiiiiii iiiiiiiiii iiiiiiiiii iiiiiiiiii |
| SCAMPI | MMMMMMMMMM MMMMMMiiii iiiiiiiiii iiiiiiiiii iiiiiiiiii |
| SPOCTOPUS | MMMMMMMMMM Miiiiiiiii iiiiiiiiii iiiiiiiiii iiiiiiiiii |
| PDB-homology |  |
|  | |
|  | 451                                         491 |
| Seq. | ADSLRSTGAP RRERDETSRA TTTTTTAAAT ATTTTRSNHR AVMSEHLNLR |
| TOPCONS | iiiiiiiiii iiiiiiiiii iiiiiiiiii iiiiiiiiii iiiiiiiiii |
| OCTOPUS | iiiiiiiiii iiiiiiiiii iiiiiiiiii iiiiiiiiii iiiiiiiiii |
| Philius | iiiiiiiiii iiiiiiiiii iiiiiiiiii iiiiiiiiii iiiiiiiiii |
| PolyPhobius | iiiiiiiiii iiiiiiiiii iiiiiiiiii iiiiiiiiii iiiiiiiiii |
| SCAMPI | iiiiiiiiii iiiiiiiiii iiiiiiiiii iiiiiiiiii iiiiiiiiii |
| SPOCTOPUS | iiiiiiiiii iiiiiiiiii iiiiiiiiii iiiiiiiiii iiiiiiiiii |
| PDB-homology |  |
|  | |
|  | 501                                         541 |
| Seq. | FVLNALGKRF RLFLVLTLLL TLIETVVSLY ELVLDFAVAA KRSSGGGGVS |
| TOPCONS | iiiiiiiiiM MMMMMMMMMM MMMMMMMMMM oooooooooo oooooooooo |
| OCTOPUS | iiiiiiiiMM MMMMMMMMMM MMMMMMMMMo oooooooooo oooooooooo |
| Philius | iiiiiiiiiM MMMMMMMMMM MMMMMMMMMM oooooooooo ooooooMMMM |
| PolyPhobius | iiiiiiiiiM MMMMMMMMMM MMMMMMMMMM MMoooooooo ooooooooMM |
| SCAMPI | iiiiiiiiiM MMMMMMMMMM MMMMMMMMMM oooooooooo oooooooooo |
| SPOCTOPUS | iiiiiiiiMM MMMMMMMMMM MMMMMMMMMo oooooooooo oooooooooo |
| PDB-homology |  |
|  | |
|  | 551                                         591 |
| Seq. | GVIAVLARAD LLLNNALHLA GIGMNVRAAT LITHRLQRVV GHFSQQHAAY |
| TOPCONS | oooooooMMM MMMMMMMMMM MMMMMMMMii iiiiiiiiii iiiiiiiiii |
| OCTOPUS | oooooooMMM MMMMMMMMMM MMMMMMMMii iiiiiiiiii iiiiiiiiii |
| Philius | MMMMMMMMMM MMMMMMMMMM MMiiiiiiii iiiiiiiiii iiiiiiiiii |
| PolyPhobius | MMMMMMMMMM MMMMMMMMii iiiiiiiiii iiiiiiiiii iiiiiiiiii |
| SCAMPI | oooooooooM MMMMMMMMMM MMMMMMMMMM iiiiiiiiii iiiiiiiiii |
| SPOCTOPUS | oooooooMMM MMMMMMMMMM MMMMMMMMii iiiiiiiiii iiiiiiiiii |
| PDB-homology |  |
|  | |
|  | 601                                         641 |
| Seq. | TMLDSDDDDD DGGDDEGGGG GDDDVENSRR RRRRSSARRR VDAFIERDAV |
| TOPCONS | iiiiiiiiii iiiiiiiiii iiiiiiiiii iiiiiiiiii iiiiiiiiii |
| OCTOPUS | iiiiiiiiii iiiiiiiiii iiiiiiiiii iiiiiiiiii iiiiiiiiii |
| Philius | iiiiiiiiii iiiiiiiiii iiiiiiiiii iiiiiiiiii iiiiiiiiii |
| PolyPhobius | iiiiiiiiii iiiiiiiiii iiiiiiiiii iiiiiiiiii iiiiiiiiii |
| SCAMPI | iiiiiiiiii iiiiiiiiii iiiiiiiiii iiiiiiiiii iiiiiiiiii |
| SPOCTOPUS | iiiiiiiiii iiiiiiiiii iiiiiiiiii iiiiiiiiii iiiiiiiiii |
| PDB-homology |  |
|  | |
|  | 651                                         691 |
| Seq. | LGHFRDRPLG ISIYGFFVDR YTIRTLDGVV FGSVIFIVSR ALMDAARADG |
| TOPCONS | iiiiiiiiii iiiiiiiiii iMMMMMMMMM MMMMMMMMMM MMoooooooo |
| OCTOPUS | iiiiiiiiii iiiiiiiiii iMMMMMMMMM MMMMMMMMMM MMoooooooo |
| Philius | iiiiiiiiii iiiiiiiiii iiiiiiiiii iiiiiiiiii iiiiiiiiii |
| PolyPhobius | iiiiiiiiii iiiiiiiiii iiiiiMMMMM MMMMMMMMMM MMMooooooo |
| SCAMPI | iiiiiiiiii iiiiiiiiii iMMMMMMMMM MMMMMMMMMM MMoooooooo |
| SPOCTOPUS | iiiiiiiiii iiiiiiiiii iMMMMMMMMM MMMMMMMMMM MMoooooooo |
| PDB-homology |  |
|  | |

|  |  |
| --- | --- |
|  | 701 |
| Seq. | E |
| TOPCONS | o |
| OCTOPUS | o |
| Philius | i |
| PolyPhobius | o |
| SCAMPI | o |
| SPOCTOPUS | o |
| PDB-homology |  |
