# Supplementary material for: A putative origin of the insect chemosensory receptor superfamily in the last common eukaryotic ancestor
Source: eLife. 2020 Dec 4;9:e62507. doi: 10.7554/eLife.62507 (PMC7746228; doi:10.7554/eLife.62507)
Supplement: Supplementary file 2. [file elife-62507-supp2.zip › 201130_SuppFile2_TOPCONS/seq_13/nicetop.html]

|  |  |
| --- | --- |
|  | 1                                           41 |
| Seq. | MTGIDRDEEP PESESNIPTA ACNAEEALRQ SAFAQISSLS VYARLQRTSG |
| TOPCONS | iiiiiiiiii iiiiiiiiii iiiiiiiiii iiiiiiiiii iiiiiiiiii |
| OCTOPUS | iiiiiiiiii iiiiiiiiii iiiiiiiiii iiiiiiiiii iiiiiiiiii |
| Philius | iiiiiiiiii iiiiiiiiii iiiiiiiiii iiiiiiiiii iiiiiiiiii |
| PolyPhobius | iiiiiiiiii iiiiiiiiii iiiiiiiiii iiiiiiiiii iiiiiiiiii |
| SCAMPI | iiiiiiiiii iiiiiiiiii iiiiiiiiii iiiiiiiiii iiiiiiiiii |
| SPOCTOPUS | iiiiiiiiii iiiiiiiiii iiiiiiiiii iiiiiiiiii iiiiiiiiii |
| PDB-homology |  |
|  | |
|  | 51                                          91 |
| Seq. | AIDDATWQRF AHQLDDLRRD MLLMHGHDDN AQNDGDNDNG ATMVDNGVST |
| TOPCONS | iiiiiiiiii iiiiiiiiii iiiiiiiiii iiiiiiiiii iiiiiiiiii |
| OCTOPUS | iiiiiiiiii iiiiiiiiii iiiiiiiiii iiiiiiiiii iiiiiiiiii |
| Philius | iiiiiiiiii iiiiiiiiii iiiiiiiiii iiiiiiiiii iiiiiiiiii |
| PolyPhobius | iiiiiiiiii iiiiiiiiii iiiiiiiiii iiiiiiiiii iiiiiiiiii |
| SCAMPI | iiiiiiiiii iiiiiiiiii iiiiiiiiii iiiiiiiiii iiiiiiiiii |
| SPOCTOPUS | iiiiiiiiii iiiiiiiiii iiiiiiiiii iiiiiiiiii iiiiiiiiii |
| PDB-homology |  |
|  | |
|  | 101                                         141 |
| Seq. | SICEPMPEPY GSQQLPTRRV VKLPSNKTAL GMGMISTQTD LHSVESSRVE |
| TOPCONS | iiiiiiiiii iiiiiiiiii iiiiiiiiii iiiiiiiiii iiiiiiiiii |
| OCTOPUS | iiiiiiiiii iiiiiiiiii iiiiiiiiii iiiiiiiiii iiiiiiiiii |
| Philius | iiiiiiiiii iiiiiiiiii iiiiiiiiii iiiiiiiiii iiiiiiiiii |
| PolyPhobius | iiiiiiiiii iiiiiiiiii iiiiiiiiii iiiiiiiiii iiiiiiiiii |
| SCAMPI | iiiiiiiiii iiiiiiiiii iiiiiiiiii iiiiiiiiii iiiiiiiiii |
| SPOCTOPUS | iiiiiiiiii iiiiiiiiii iiiiiiiiii iiiiiiiiii iiiiiiiiii |
| PDB-homology |  |
|  | |
|  | 151                                         191 |
| Seq. | VGWVQWLMMM FLHPKHFENL TPALEDVVDF MVHGTPLETW WVRIAGLSFY |
| TOPCONS | iiiiiiiiii iiiiiiiiii iiiiiiiiii iiiiiiiiii iiiiiiiiii |
| OCTOPUS | iiiiiiiiii iiiiiiiiii iiiiiiiiii iiiiiiiiii iiiiiiiiii |
| Philius | iiiiiiiiii iiiiiiiiii iiiiiiiiii iiiiiiiiii iiiiiiiiii |
| PolyPhobius | iiiiiiiiii iiiiiiiiii iiiiiiiiii iiiiiiiiii iiiiiiiiii |
| SCAMPI | iiiiiiiiii iiiiiiiiii iiiiiiiiii iiiiiiiiii iiiiiiiiii |
| SPOCTOPUS | iiiiiiiiii iiiiiiiiii iiiiiiiiii iiiiiiiiii iiiiiiiiii |
| PDB-homology |  |
|  | |
|  | 201                                         241 |
| Seq. | PHDSRLVRQI WPIMIHLILW HGAICNGVVF FFSAIDPMWC DRNYDSDLCE |
| TOPCONS | iiiiiiiMMM MMMMMMMMMM MMMMMMMMoo oooooooooo oooooooooo |
| OCTOPUS | iiiiiiiMMM MMMMMMMMMM MMMMMMMMoo oooooooooo oooooooooo |
| Philius | iiiiiiiiii iiiMMMMMMM MMMMMMMMMM MMMMMooooo oooooooooo |
| PolyPhobius | iiiiiiiiii iiMMMMMMMM MMMMMMMMMM MMMMMooooo oooooooooo |
| SCAMPI | iiiiMMMMMM MMMMMMMMMM MMMMMooooo oooooooooo oooooooooo |
| SPOCTOPUS | iiiiiiiMMM MMMMMMMMMM MMMMMMMMoo oooooooooo oooooooooo |
| PDB-homology |  |
|  | |
|  | 251                                         291 |
| Seq. | GTDLLLLFMF WSISLAVAVA YTVLRVQWIY RGDAFLHALD FVRSECNEAN |
| TOPCONS | oooMMMMMMM MMMMMMMMMM MMMMiiiiii iiiiiiiiii iiiiiiiiii |
| OCTOPUS | ooooMMMMMM MMMMMMMMMM MMMMMiiiii iiiiiiiiii iiiiiiiiii |
| Philius | oooMMMMMMM MMMMMMMMMM MMMMiiiiii iiiiiiiiii iiiiiiiiii |
| PolyPhobius | oooMMMMMMM MMMMMMMMMM MMMMiiiiii iiiiiiiiii iiiiiiiiii |
| SCAMPI | oooMMMMMMM MMMMMMMMMM MMMMiiiiii iiiiiiiiii iiiiiiiiii |
| SPOCTOPUS | oooooMMMMM MMMMMMMMMM MMMMMMiiii iiiiiiiiii iiiiiiiiii |
| PDB-homology |  |
|  | |
|  | 301                                         341 |
| Seq. | VLHRLKKDVR AKFAWTVVFT FLFWSYFFYI GVIDRALVLS DRHKKYFAIF |
| TOPCONS | iiiiiiiiii iiMMMMMMMM MMMMMMMMMM MMMooooooo oooooooooo |
| OCTOPUS | iiiiiiiiii MMMMMMMMMM MMMMMMMMMM Mooooooooo oooooooooo |
| Philius | iiiiiiiiii iiMMMMMMMM MMMMMMMMMM MMMooooooo oooooooooo |
| PolyPhobius | iiiiiiiiii iiMMMMMMMM MMMMMMMMMM MMMooooooo oooooooooo |
| SCAMPI | iiiiiiiiii iiMMMMMMMM MMMMMMMMMM MMMooooooo oooooooooo |
| SPOCTOPUS | iiiiiiiiii MMMMMMMMMM MMMMMMMMMM Mooooooooo oooooooooo |
| PDB-homology |  |
|  | |
|  | 351                                         391 |
| Seq. | PGMHNAPQWL LELNGYLLEI CGTLFEPFIG MTVAAQTGII CVIHRSSFNV |
| TOPCONS | oooooooooo oooooooooo ooMMMMMMMM MMMMMMMMMM MMMiiiiiii |
| OCTOPUS | oooooooooo ooooooooMM MMMMMMMMMM MMMMMMMMMi iiiiiiiiii |
| Philius | oooooooooo oooooooooo oooooooMMM MMMMMMMMMM MMMMMMMMii |
| PolyPhobius | oooooooooo oooooooooo ooooooooMM MMMMMMMMMM MMMMMMMMMM |
| SCAMPI | oooooooooo oooooooooo ooMMMMMMMM MMMMMMMMMM MMMiiiiiii |
| SPOCTOPUS | oooooooooo ooooooooMM MMMMMMMMMM MMMMMMMMMi iiiiiiiiii |
| PDB-homology |  |
|  | |
|  | 401                                         441 |
| Seq. | LMYHMMKRDR HMQMHARAQE YSSRPPMAMA LTVPQLIEMH RSLDGMLNRS |
| TOPCONS | iiiiiiiiii iiiiiiiiii iiiiiiiiii iiiiiiiiii iiiiiiiiii |
| OCTOPUS | iiiiiiiiii iiiiiiiiii iiiiiiiiii iiiiiiiiii iiiiiiiiii |
| Philius | iiiiiiiiii iiiiiiiiii iiiiiiiiii iiiiiiiiii iiiiiiiiii |
| PolyPhobius | MMMiiiiiii iiiiiiiiii iiiiiiiiii iiiiiiiiii iiiiiiiiii |
| SCAMPI | iiiiiiiiii iiiiiiiiii iiiiiiiiii iiiiiiiiii iiiiiiiiii |
| SPOCTOPUS | iiiiiiiiii iiiiiiiiii iiiiiiiiii iiiiiiiiii iiiiiiiiii |
| PDB-homology |  |
|  | |
|  | 451                                         491 |
| Seq. | SLILQAPITA MGALYFVSFL SCLFLILFRE PSSNGLDVFF IVLGIFFTVG |
| TOPCONS | iiiMMMMMMM MMMMMMMMMM MMMMoooooo ooooooMMMM MMMMMMMMMM |
| OCTOPUS | iiiMMMMMMM MMMMMMMMMM MMMMoooooo ooooooMMMM MMMMMMMMMM |
| Philius | iiiiiMMMMM MMMMMMMMMM MMMMMMMMoo oooooMMMMM MMMMMMMMMM |
| PolyPhobius | iiMMMMMMMM MMMMMMMMMM MMMMMMMooo oooooooMMM MMMMMMMMMM |
| SCAMPI | iiMMMMMMMM MMMMMMMMMM MMMooooooo ooooooMMMM MMMMMMMMMM |
| SPOCTOPUS | iiiiMMMMMM MMMMMMMMMM MMMMMooooo ooooooMMMM MMMMMMMMMM |
| PDB-homology |  |
|  | |
|  | 501                                         541 |
| Seq. | LSTYWILSDT AKVTAKCARL AEIASFASRH YYLCGGTENR PSLPTPASSH |
| TOPCONS | MMMMMMMiii iiiiiiiiii iiiiiiiiii iiiiiiiiii iiiiiiiiii |
| OCTOPUS | MMMMMMMiii iiiiiiiiii iiiiiiiiii iiiiiiiiii iiiiiiiiii |
| Philius | MMMMMMMiii iiiiiiiiii iiiiiiiiii iiiiiiiiii iiiiiiiiii |
| PolyPhobius | MMMMMMMiii iiiiiiiiii iiiiiiiiii iiiiiiiiii iiiiiiiiii |
| SCAMPI | MMMMMMMiii iiiiiiiiii iiiiiiiiii iiiiiiiiii iiiiiiiiii |
| SPOCTOPUS | MMMMMMMiii iiiiiiiiii iiiiiiiiii iiiiiiiiii iiiiiiiiii |
| PDB-homology |  |
|  | |
|  | 551                                         591 |
| Seq. | APTHAIVRDL TLSKAVADSI MLAAATPRSS EAPSRQATAD LSITFPTNGS |
| TOPCONS | iiiiiiiiii iiiiiiiiii iiiiiiiiii iiiiiiiiii iiiiiiiiii |
| OCTOPUS | iiiiiiiiii iiiiiiiiii iiiiiiiiii iiiiiiiiii iiiiiiiiii |
| Philius | iiiiiiiiii iiiiiiiiii iiiiiiiiii iiiiiiiiii iiiiiiiiii |
| PolyPhobius | iiiiiiiiii iiiiiiiiii iiiiiiiiii iiiiiiiiii iiiiiiiiii |
| SCAMPI | iiiiiiiiii iiiiiiiiii iiiiiiiiii iiiiiiiiii iiiiiiiiii |
| SPOCTOPUS | iiiiiiiiii iiiiiiiiii iiiiiiiiii iiiiiiiiii iiiiiiiiii |
| PDB-homology |  |
|  | |
|  | 601                                         641 |
| Seq. | TVAAPEHSRT NIKRSFFVLR PSVARLMRAW RRLRKVKSAE PRKIEIQSRS |
| TOPCONS | iiiiiiiiii iiiiiiiiii iiiiiiiiii iiiiiiiiii iiiiiiiiii |
| OCTOPUS | iiiiiiiiii iiiiiiiiii iiiiiiiiii iiiiiiiiii iiiiiiiiii |
| Philius | iiiiiiiiii iiiiiiiiii iiiiiiiiii iiiiiiiiii iiiiiiiiii |
| PolyPhobius | iiiiiiiiii iiiiiiiiii iiiiiiiiii iiiiiiiiii iiiiiiiiii |
| SCAMPI | iiiiiiiiii iiiiiiiiii iiiiiiiiii iiiiiiiiii iiiiiiiiii |
| SPOCTOPUS | iiiiiiiiii iiiiiiiiii iiiiiiiiii iiiiiiiiii iiiiiiiiii |
| PDB-homology |  |
|  | |
|  | 651                                         691 |
| Seq. | RRAGEEDLES GVLGDIPVRD DSCALSYTAF VRESDAGLGG VCDGMGMGGG |
| TOPCONS | iiiiiiiiii iiiiiiiiii iiiiiiiiii iiiiiiiiii iiiiiiiiii |
| OCTOPUS | iiiiiiiiii iiiiiiiiii iiiiiiiiii iiiiiiiiii iiiiiiiiii |
| Philius | iiiiiiiiii iiiiiiiiii iiiiiiiiii iiiiiiiiii iiiiiiiiii |
| PolyPhobius | iiiiiiiiii iiiiiiiiii iiiiiiiiii iiiiiiiiii iiiiiiiiii |
| SCAMPI | iiiiiiiiii iiiiiiiiii iiiiiiiiii iiiiiiiiii iiiiiiiiii |
| SPOCTOPUS | iiiiiiiiii iiiiiiiiii iiiiiiiiii iiiiiiiiii iiiiiiiiii |
| PDB-homology |  |
|  | |
|  | 701                                         741 |
| Seq. | GGVYNVGRPG VKPASSGCDI MHEAMEQLLL VQYFRASNSA WRVYGVEMTS |
| TOPCONS | iiiiiiiiii iiiiiiiiii iiiiiiiiii iiiiiiiiii iiiiiiiiii |
| OCTOPUS | iiiiiiiiii iiiiiiiiii iiiiiiiiii iiiiiiiiii iiiiiiiiii |
| Philius | iiiiiiiiii iiiiiiiiii iiiiiiiiii iiiiiiiiii iiiiiiiiii |
| PolyPhobius | iiiiiiiiii iiiiiiiiii iiiiiiiiii iiiiiiiiii iiiiiiiiii |
| SCAMPI | iiiiiiiiii iiiiiiiiii iiiiiiiiii iiiiiiiiii iiiiiiiiii |
| SPOCTOPUS | iiiiiiiiii iiiiiiiiii iiiiiiiiii iiiiiiiiii iiiiiiiiii |
| PDB-homology |  |
|  | |

|  |  |
| --- | --- |
|  | 751                   771 |
| Seq. | SLLGRILYTV AALVAVGLQR ALSL |
| TOPCONS | iMMMMMMMMM MMMMMMMMMM MMoo |
| OCTOPUS | iiiiiiiiii iiiiiiiiii iiii |
| Philius | MMMMMMMMMM MMMMMMMMMM MMoo |
| PolyPhobius | iMMMMMMMMM MMMMMMMMMM MMoo |
| SCAMPI | iMMMMMMMMM MMMMMMMMMM MMoo |
| SPOCTOPUS | iiiiiiiiii iiiiiiiiii iiii |
| PDB-homology |  |
