# Supplementary material for: A putative origin of the insect chemosensory receptor superfamily in the last common eukaryotic ancestor
Source: eLife. 2020 Dec 4;9:e62507. doi: 10.7554/eLife.62507 (PMC7746228; doi:10.7554/eLife.62507)
Supplement: Supplementary file 2. [file elife-62507-supp2.zip › 201130_SuppFile2_TOPCONS/seq_14/nicetop.html]

|  |  |
| --- | --- |
|  | 1                                           41 |
| Seq. | MPEDPENASP APVAASAPAR EAALHAVVRS QLDALQNTAD ALTLVKTTQE |
| TOPCONS | iiiiiiiiii iiiiiiiiii iiiiiiiiii iiiiiiiiii iiiiiiiiii |
| OCTOPUS | iiiiiiiiii iiiiiiiiii iiiiiiiiii iiiiiiiiii iiiiiiiiii |
| Philius | iiiiiiiiii iiiiiiiiii iiiiiiiiii iiiiiiiiii iiiiiiiiii |
| PolyPhobius | iiiiiiiiii iiiiiiiiii iiiiiiiiii iiiiiiiiii iiiiiiiiii |
| SCAMPI | iiiiiiiiii iiiiiiiiii iiiiiiiiii iiiiiiiiii iiiiiiiiii |
| SPOCTOPUS | iiiiiiiiii iiiiiiiiii iiiiiiiiii iiiiiiiiii iiiiiiiiii |
| PDB-homology |  |
|  | |
|  | 51                                          91 |
| Seq. | AIQTSIVQLR ELFKASPASE RPRARTLAPQ LPTLTRRVAR APSAPSADVP |
| TOPCONS | iiiiiiiiii iiiiiiiiii iiiiiiiiii iiiiiiiiii iiiiiiiiii |
| OCTOPUS | iiiiiiiiii iiiiiiiiii iiiiiiiiii iiiiiiiiii iiiiiiiiii |
| Philius | iiiiiiiiii iiiiiiiiii iiiiiiiiii iiiiiiiiii iiiiiiiiii |
| PolyPhobius | iiiiiiiiii iiiiiiiiii iiiiiiiiii iiiiiiiiii iiiiiiiiii |
| SCAMPI | iiiiiiiiii iiiiiiiiii iiiiiiiiii iiiiiiiiii iiiiiiiiii |
| SPOCTOPUS | iiiiiiiiii iiiiiiiiii iiiiiiiiii iiiiiiiiii iiiiiiiiii |
| PDB-homology |  |
|  | |
|  | 101                                         141 |
| Seq. | GPANGFKQAP SAGIRQRSIT INGIGATHPT AASTPDTGDE EHPALQDAPY |
| TOPCONS | iiiiiiiiii iiiiiiiiii iiiiiiiiii iiiiiiiiii iiiiiiiiii |
| OCTOPUS | iiiiiiiiii iiiiiiiiii iiiiiiiiii iiiiiiiiii iiiiiiiiii |
| Philius | iiiiiiiiii iiiiiiiiii iiiiiiiiii iiiiiiiiii iiiiiiiiii |
| PolyPhobius | iiiiiiiiii iiiiiiiiii iiiiiiiiii iiiiiiiiii iiiiiiiiii |
| SCAMPI | iiiiiiiiii iiiiiiiiii iiiiiiiiii iiiiiiiiii iiiiiiiiii |
| SPOCTOPUS | iiiiiiiiii iiiiiiiiii iiiiiiiiii iiiiiiiiii iiiiiiiiii |
| PDB-homology |  |
|  | |
|  | 151                                         191 |
| Seq. | SRALGTRTES VASYESSTEG EGALRAFCRH YWQGILEPRM PVETWETAKM |
| TOPCONS | iiiiiiiiii iiiiiiiiii iiiiiiiiii iiiiiiiiii iiiiiiiiii |
| OCTOPUS | iiiiiiiiii iiiiiiiiii iiiiiiiiii iiiiiiiiii iiiiiiiiii |
| Philius | iiiiiiiiii iiiiiiiiii iiiiiiiiii iiiiiiiiii iiiiiiiiii |
| PolyPhobius | iiiiiiiiii iiiiiiiiii iiiiiiiiii iiiiiiiiii iiiiiiiiii |
| SCAMPI | iiiiiiiiii iiiiiiiiii iiiiiiiiii iiiiiiiiii iiiiiiiiii |
| SPOCTOPUS | iiiiiiiiii iiiiiiiiii iiiiiiiiii iiiiiiiiii iiiiiiiiii |
| PDB-homology |  |
|  | |
|  | 201                                         241 |
| Seq. | LRKGNKVLAV WARLAGLSFI PDDGVFLLVI YPLIIQLILW HSAVMGIISF |
| TOPCONS | iiiiiiiiii iiiiiiiiii iiiiiiiMMM MMMMMMMMMM MMMMMMMMoo |
| OCTOPUS | iiiiiiiiii iiiiiiiiii iiiiiiiMMM MMMMMMMMMM MMMMMMMMoo |
| Philius | iiiiiiiiMM MMMMMMMMMM MMMMMMMMMM MoooooMMMM MMMMMMMMMM |
| PolyPhobius | iiiiiiiMMM MMMMMMMMMM MMMMMMMMMM MoooooMMMM MMMMMMMMMM |
| SCAMPI | iiiiiiiiii iiiiiiiiii iiiiMMMMMM MMMMMMMMMM MMMMMooooo |
| SPOCTOPUS | iiiiiiiiii iiiiiiiiii iiiiiiiMMM MMMMMMMMMM MMMMMMMMoo |
| PDB-homology |  |
|  | |
|  | 251                                         291 |
| Seq. | TSSVIGGSKV MIQAGDPVVQ FLFLILMFAI ALSYTVLRAQ WVTRGYTFLS |
| TOPCONS | oooooooooo oooooooMMM MMMMMMMMMM MMMMMMMMii iiiiiiiiii |
| OCTOPUS | oooooooooo oooooooMMM MMMMMMMMMM MMMMMMMMii iiiiiiiiii |
| Philius | MMMMMMMiii iiiiiiiMMM MMMMMMMMMM MMMMMMMooo ooooMMMMMM |
| PolyPhobius | MMMMMMiiii iiiiiiiMMM MMMMMMMMMM MMMMMMMooo ooMMMMMMMM |
| SCAMPI | oooooooooo ooooooMMMM MMMMMMMMMM MMMMMMMiii iiiiiiiiii |
| SPOCTOPUS | oooooooooo ooooooooMM MMMMMMMMMM MMMMMMMMMi iiiiiiiiii |
| PDB-homology |  |
|  | |
|  | 301                                         341 |
| Seq. | AATFVLENGV DLARKVKGMA RVRFAVCLSF AFLCTLVVIV FNQQVFATFI |
| TOPCONS | iiiiiiiiii iiiiiiiiii iMMMMMMMMM MMMMMMMMMM MMoooooooo |
| OCTOPUS | iiiiiiiiii iiiiiiiiii iMMMMMMMMM MMMMMMMMMM MMoooooooo |
| Philius | MMMMMMMMMM MMiiiiiiii iiiMMMMMMM MMMMMMMMMM MMMMMMoooo |
| PolyPhobius | MMMMMMMMMM iiiiiiiiii iMMMMMMMMM MMMMMMMMMM MMMMoooooo |
| SCAMPI | iiiiiiiiii iiiiiiiiii iMMMMMMMMM MMMMMMMMMM MMoooooooo |
| SPOCTOPUS | iiiiiiiiii iiiiiiiiii iMMMMMMMMM MMMMMMMMMM MMoooooooo |
| PDB-homology |  |
|  | |
|  | 351                                         391 |
| Seq. | FVENGSFAWK VNVWISVVLG HFFGPFCGLT VAGIMADICD IHSATVNVLL |
| TOPCONS | oooooooooo ooooooooMM MMMMMMMMMM MMMMMMMMMi iiiiiiiiii |
| OCTOPUS | oooooooooo ooooooooMM MMMMMMMMMM MMMMMMMMMi iiiiiiiiii |
| Philius | oooooooooo ooMMMMMMMM MMMMMMMMMM MMMMMMiiii iiiiiiiiii |
| PolyPhobius | oooooooooo ooMMMMMMMM MMMMMMMMMM MMMMMiiiii iiiiiiiiii |
| SCAMPI | oooooooooo oooooooooo MMMMMMMMMM MMMMMMMMMM Miiiiiiiii |
| SPOCTOPUS | oooooooooo ooooooooMM MMMMMMMMMM MMMMMMMMMi iiiiiiiiii |
| PDB-homology |  |
|  | |
|  | 401                                         441 |
| Seq. | RRLTERAQSK NKTKTKTKAL DAYQLIALHR RVDGFLVRSA RVLELPITVQ |
| TOPCONS | iiiiiiiiii iiiiiiiiii iiiiiiiiii iiiiiiiiii iiiMMMMMMM |
| OCTOPUS | iiiiiiiiii iiiiiiiiii iiiiiiiiii iiiiiiiiii iiiMMMMMMM |
| Philius | iiiiiiiiii iiiiiiiiii iiiiiiiiii iiiiiiiiii iiiiiiMMMM |
| PolyPhobius | iiiiiiiiii iiiiiiiiii iiiiiiiiii iiiiiiiiii iiiiMMMMMM |
| SCAMPI | iiiiiiiiii iiiiiiiiii iiiiiiiiii iiiiiiiiii iMMMMMMMMM |
| SPOCTOPUS | iiiiiiiiii iiiiiiiiii iiiiiiiiii iiiiiiiiii iiiMMMMMMM |
| PDB-homology |  |
|  | |
|  | 451                                         491 |
| Seq. | TTLFFVCFLT CAFILIFHKG RGSTGDPEED AESVAVYHVA PLVIFVAVSV |
| TOPCONS | MMMMMMMMMM MMMMoooooo oooooooooo oooooMMMMM MMMMMMMMMM |
| OCTOPUS | MMMMMMMMMM MMMMoooooo oooooooooo ooooooMMMM MMMMMMMMMM |
| Philius | MMMMMMMMMM MMMMMMMooo oooooooooo ooooMMMMMM MMMMMMMMMM |
| PolyPhobius | MMMMMMMMMM MMMMMMMooo oooooooooo oooooMMMMM MMMMMMMMMM |
| SCAMPI | MMMMMMMMMM MMoooooooo oooooooooo oooooMMMMM MMMMMMMMMM |
| SPOCTOPUS | MMMMMMMMMM MMMMoooooo oooooooooo ooooooMMMM MMMMMMMMMM |
| PDB-homology |  |
|  | |
|  | 501                                         541 |
| Seq. | ANYWILSSSS GVTMRCRRLP VIASLSCRSP RPPSLGTTPP GSRPDDANVH |
| TOPCONS | MMMMMMiiii iiiiiiiiii iiiiiiiiii iiiiiiiiii iiiiiiiiii |
| OCTOPUS | MMMMMMMiii iiiiiiiiii iiiiiiiiii iiiiiiiiii iiiiiiiiii |
| Philius | MMMMMMiiii iiiiiiiiii iiiiiiiiii iiiiiiiiii iiiiiiiiii |
| PolyPhobius | MMMMMMiiii iiiiiiiiii iiiiiiiiii iiiiiiiiii iiiiiiiiii |
| SCAMPI | MMMMMMiiii iiiiiiiiii iiiiiiiiii iiiiiiiiii iiiiiiiiii |
| SPOCTOPUS | MMMMMMMiii iiiiiiiiii iiiiiiiiii iiiiiiiiii iiiiiiiiii |
| PDB-homology |  |
|  | |
|  | 551                                         591 |
| Seq. | SPPLLRSRTL TKADAIREKL DVIQEEGIVA SSNLDILEGR AAKSEEVRKA |
| TOPCONS | iiiiiiiiii iiiiiiiiii iiiiiiiiii iiiiiiiiii iiiiiiiiii |
| OCTOPUS | iiiiiiiiii iiiiiiiiii iiiiiiiiii iiiiiiiiii iiiiiiiiii |
| Philius | iiiiiiiiii iiiiiiiiii iiiiiiiiii iiiiiiiiii iiiiiiiiii |
| PolyPhobius | iiiiiiiiii iiiiiiiiii iiiiiiiiii iiiiiiiiii iiiiiiiiii |
| SCAMPI | iiiiiiiiii iiiiiiiiii iiiiiiiiii iiiiiiiiii iiiiiiiiii |
| SPOCTOPUS | iiiiiiiiii iiiiiiiiii iiiiiiiiii iiiiiiiiii iiiiiiiiii |
| PDB-homology |  |
|  | |
|  | 601                                         641 |
| Seq. | TWWRIMKGLF PSKSERQQQK RRVRSIDIEW REMATRDNYD TDNDGADDGC |
| TOPCONS | iiiiiiiiii iiiiiiiiii iiiiiiiiii iiiiiiiiii iiiiiiiiii |
| OCTOPUS | iiiiiiiiii iiiiiiiiii iiiiiiiiii iiiiiiiiii iiiiiiiiii |
| Philius | iiiiiiiiii iiiiiiiiii iiiiiiiiii iiiiiiiiii iiiiiiiiii |
| PolyPhobius | iiiiiiiiii iiiiiiiiii iiiiiiiiii iiiiiiiiii iiiiiiiiii |
| SCAMPI | iiiiiiiiii iiiiiiiiii iiiiiiiiii iiiiiiiiii iiiiiiiiii |
| SPOCTOPUS | iiiiiiiiii iiiiiiiiii iiiiiiiiii iiiiiiiiii iiiiiiiiii |
| PDB-homology |  |
|  | |
|  | 651                                         691 |
| Seq. | QLPPAETPVA AVRVASSKSL ASILVDHLPL GPPGVSEAET RPSTTLRSMS |
| TOPCONS | iiiiiiiiii iiiiiiiiii iiiiiiiiii iiiiiiiiii iiiiiiiiii |
| OCTOPUS | iiiiiiiiii iiiiiiiiii iiiiiiiiii iiiiiiiiii iiiiiiiiii |
| Philius | iiiiiiiiii iiiiiiiiii iiiiiiiiii iiiiiiiiii iiiiiiiiii |
| PolyPhobius | iiiiiiiiii iiiiiiiiii iiiiiiiiii iiiiiiiiii iiiiiiiiii |
| SCAMPI | iiiiiiiiii iiiiiiiiii iiiiiiiiii iiiiiiiiii iiiiiiiiii |
| SPOCTOPUS | iiiiiiiiii iiiiiiiiii iiiiiiiiii iiiiiiiiii iiiiiiiiii |
| PDB-homology |  |
|  | |
|  | 701                                         741 |
| Seq. | RQSTEESNVA PSRGGGAGAG GLGEEAARQY VDDVAEQLLL IQYFTAANSG |
| TOPCONS | iiiiiiiiii iiiiiiiiii iiiiiiiiii iiiiiiiiii iiiiiiiiii |
| OCTOPUS | iiiiiiiiii iiiiiiiiii iiiiiiiiii iiiiiiiiii iiiiiiiiii |
| Philius | iiiiiiiiii iiiiiiiiii iiiiiiiiii iiiiiiiiii iiiiiiiiii |
| PolyPhobius | iiiiiiiiii iiiiiiiiii iiiiiiiiii iiiiiiiiii iiiiiiiiii |
| SCAMPI | iiiiiiiiii iiiiiiiiii iiiiiiiiii iiiiiiiiii iiiiiiiiii |
| SPOCTOPUS | iiiiiiiiii iiiiiiiiii iiiiiiiiii iiiiiiiiii iiiiiiiiii |
| PDB-homology |  |
|  | |

|  |  |
| --- | --- |
|  | 751                              781 |
| Seq. | WRVYDVLVTT DLTGRVLYGI LTVLAFALQR TVFTDMYS |
| TOPCONS | iiiiiiiiii iiiiiiiiii iiiiiiiiii iiiiiiii |
| OCTOPUS | iiiiiiiiii iiiiiiiiii iiiiiiiiii iiiiiiii |
| Philius | iiiiiiiiii iiiiiiiiii iiiiiiiiii iiiiiiii |
| PolyPhobius | iiiiiiiiii iiiiiMMMMM MMMMMMMMMM MMMooooo |
| SCAMPI | iiiiiiiiii iMMMMMMMMM MMMMMMMMMM MMoooooo |
| SPOCTOPUS | iiiiiiiiii iiiiiiiiii iiiiiiiiii iiiiiiii |
| PDB-homology |  |
