# Supplementary material for: A putative origin of the insect chemosensory receptor superfamily in the last common eukaryotic ancestor
Source: eLife. 2020 Dec 4;9:e62507. doi: 10.7554/eLife.62507 (PMC7746228; doi:10.7554/eLife.62507)
Supplement: Supplementary file 2. [file elife-62507-supp2.zip › 201130_SuppFile2_TOPCONS/seq_15/nicetop.html]

|  |  |
| --- | --- |
|  | 1                                           41 |
| Seq. | MGSTMRSDFY TTLDQESSLA KPLVSSRSMA KVYFTHNVGT SRLSQLMRLP |
| TOPCONS | iiiiiiiiii iiiiiiiiii iiiiiiiiii iiiiiiiiii iiiiiiiiii |
| OCTOPUS | iiiiiiiiii iiiiiiiiii iiiiiiiiii iiiiiiiiii iiiiiiiiii |
| Philius | iiiiiiiiii iiiiiiiiii iiiiiiiiii iiiiiiiiii iiiiiiiiii |
| PolyPhobius | iiiiiiiiii iiiiiiiiii iiiiiiiiii iiiiiiiiii iiiiiiiiii |
| SCAMPI | iiiiiiiiii iiiiiiiiii iiiiiiiiii iiiiiiiiii iiiiiiiiii |
| SPOCTOPUS | iiiiiiiiii iiiiiiiiii iiiiiiiiii iiiiiiiiii iiiiiiiiii |
| PDB-homology |  |
|  | |
|  | 51                                          91 |
| Seq. | RFQTSRVKHP EEGGGFLYPT RTRALKAELQ RASDYFATIL AFLGFRPGDR |
| TOPCONS | iiiiiiiiii iiiiiiiiii iiiiiiiiii iiiiiiiiii iiiiiiiiii |
| OCTOPUS | iiiiiiiiii iiiiiiiiii iiiiiiiiii iiiiiiiiii iiiiiiiiii |
| Philius | iiiiiiiiii iiiiiiiiii iiiiiiiiii iiiiiiiiii iiiiiiiiii |
| PolyPhobius | iiiiiiiiii iiiiiiiiii iiiiiiiiii iiiiiiiiii iiiiiiiiii |
| SCAMPI | iiiiiiiiii iiiiiiiiii iiiiiiiiii iiiiiiiiii iiiiiiiiii |
| SPOCTOPUS | iiiiiiiiii iiiiiiiiii iiiiiiiiii iiiiiiiiii iiiiiiiiii |
| PDB-homology |  |
|  | |
|  | 101                                         141 |
| Seq. | KRFGLLFTLV SMLFLGFLFP IFDMICSDDV VSDQKLKGLI HMTGIYRFLV |
| TOPCONS | iiMMMMMMMM MMMMMMMMMM MMMooooooo oooooooooo ooooMMMMMM |
| OCTOPUS | iiMMMMMMMM MMMMMMMMMM MMMooooooo oooooooooo ooooMMMMMM |
| Philius | iiMMMMMMMM MMMMMMMMMM MMoooooooo ooooooooMM MMMMMMMMMM |
| PolyPhobius | iiMMMMMMMM MMMMMMMMMM MMMooooooo ooooooooMM MMMMMMMMMM |
| SCAMPI | iiiiMMMMMM MMMMMMMMMM MMMMMooooo oooooooooo ooooMMMMMM |
| SPOCTOPUS | iiiMMMMMMM MMMMMMMMMM MMMMoooooo oooooooooo ooooMMMMMM |
| PDB-homology |  |
|  | |
|  | 151                                         191 |
| Seq. | YIVSLLWMLR VSRRWNLDAL LFLKVDKLYV SRIAEEKGLG GMWVYAAIIQ |
| TOPCONS | MMMMMMMMMM MMMMMiiiii iiiiiiiiii iiiiiiiiii iiiiiiiiii |
| OCTOPUS | MMMMMMMMMM MMMMMiiiii iiiiiiiiii iiiiiiiiii iiiiiiiiii |
| Philius | MMMMMMMMMi iiiiiiiiii iiiiiiiiii iiiiiiiiii iiiiiiiiii |
| PolyPhobius | MMMMMMMMMi iiiiiiiiii iiiiiiiiii iiiiiiiiii iiiiiiiiii |
| SCAMPI | MMMMMMMMMM MMMMMiiiii iiiiiiiiii iiiiiiiiii iiiiiiiiii |
| SPOCTOPUS | MMMMMMMMMM MMMMMiiiii iiiiiiiiii iiiiiiiiii iiiiiiiiii |
| PDB-homology |  |
|  | |
|  | 201                                         241 |
| Seq. | NFALEVMCLF GPYFLIIVFA KAWWFFNSLG NSKLYSSSYN FMGHVLMDSS |
| TOPCONS | MMMMMMMMMM MMMMMMMMMM Mooooooooo oooooooooo oooooooooo |
| OCTOPUS | MMMMMMMMMM MMMMMMMMMM Mooooooooo oooooooooo oooooooooo |
| Philius | MMMMMMMMMM MMMMMMMMMM MMMMoooooo oooooooooo oooooooooo |
| PolyPhobius | iMMMMMMMMM MMMMMMMMMM MMMMMMoooo oooooooooo oooooooooo |
| SCAMPI | iiiMMMMMMM MMMMMMMMMM MMMMoooooo oooooooooo oooooooooo |
| SPOCTOPUS | MMMMMMMMMM MMMMMMMMMM Mooooooooo oooooooooo oooooooooo |
| PDB-homology |  |
|  | |
|  | 251                                         291 |
| Seq. | SPLYVKILFY FADLLSQSYM QIIYLVPCVY FRMVSSLVLL EMKAYKEMVT |
| TOPCONS | oooooooooo oooooooooM MMMMMMMMMM MMMMMMMMMM iiiiiiiiii |
| OCTOPUS | oooooooooM MMMMMMMMMM MMMMMMMMMM MMMMMMMMMM iiiiiiiiii |
| Philius | oooooooooo oooooooMMM MMMMMMMMMM MMMMMMMMMM iiiiiiiiii |
| PolyPhobius | oooooooooo ooooooooMM MMMMMMMMMM MMMMMMMMMM iiiiiiiiii |
| SCAMPI | oooooooooo oooooooooo oMMMMMMMMM MMMMMMMMMM MMiiiiiiii |
| SPOCTOPUS | oooooooooM MMMMMMMMMM MMMMMMMMMM MMMMMMMMMM iiiiiiiiii |
| PDB-homology |  |
|  | |
|  | 301                                         341 |
| Seq. | PDKKADEKND QLGELLSEDV SMQHSLASNG SAATVERGNS FSASKGLNAS |
| TOPCONS | iiiiiiiiii iiiiiiiiii iiiiiiiiii iiiiiiiiii iiiiiiiiii |
| OCTOPUS | iiiiiiiiii iiiiiiiiii iiiiiiiiii iiiiiiiiii iiiiiiiiii |
| Philius | iiiiiiiiii iiiiiiiiii iiiiiiiiii iiiiiiiiii iiiiiiiiii |
| PolyPhobius | iiiiiiiiii iiiiiiiiii iiiiiiiiii iiiiiiiiii iiiiiiiiii |
| SCAMPI | iiiiiiiiii iiiiiiiiii iiiiiiiiii iiiiiiiiii iiiiiiiiii |
| SPOCTOPUS | iiiiiiiiii iiiiiiiiii iiiiiiiiii iiiiiiiiii iiiiiiiiii |
| PDB-homology |  |
|  | |
|  | 351                                         391 |
| Seq. | SWCNSSMDAK KGDDEESAKD PNLEDSMDKF ELEDLPSPSH RDAEAGIQRG |
| TOPCONS | iiiiiiiiii iiiiiiiiii iiiiiiiiii iiiiiiiiii iiiiiiiiii |
| OCTOPUS | iiiiiiiiii iiiiiiiiii iiiiiiiiii iiiiiiiiii iiiiiiiiii |
| Philius | iiiiiiiiii iiiiiiiiii iiiiiiiiii iiiiiiiiii iiiiiiiiii |
| PolyPhobius | iiiiiiiiii iiiiiiiiii iiiiiiiiii iiiiiiiiii iiiiiiiiii |
| SCAMPI | iiiiiiiiii iiiiiiiiii iiiiiiiiii iiiiiiiiii iiiiiiiiii |
| SPOCTOPUS | iiiiiiiiii iiiiiiiiii iiiiiiiiii iiiiiiiiii iiiiiiiiii |
| PDB-homology |  |
|  | |
|  | 401                                         441 |
| Seq. | PPGGLNLPNK VYMSKNAMGA MRRRRSSVYD RYGSNPFGLY VDSSKRIPED |
| TOPCONS | iiiiiiiiii iiiiiiiiii iiiiiiiiii iiiiiiiiii iiiiiiiiii |
| OCTOPUS | iiiiiiiiii iiiiiiiiii iiiiiiiiii iiiiiiiiii iiiiiiiiii |
| Philius | iiiiiiiiii iiiiiiiiii iiiiiiiiii iiiiiiiiii iiiiiiiiii |
| PolyPhobius | iiiiiiiiii iiiiiiiiii iiiiiiiiii iiiiiiiiii iiiiiiiiii |
| SCAMPI | iiiiiiiiii iiiiiiiiii iiiiiiiiii iiiiiiiiii iiiiiiiiii |
| SPOCTOPUS | iiiiiiiiii iiiiiiiiii iiiiiiiiii iiiiiiiiii iiiiiiiiii |
| PDB-homology |  |
|  | |
|  | 451                                         491 |
| Seq. | DMYEFTDSEE EDEMKHHHDD EQSFPVLREH SLLQKVLRLI SHRFRQFLLA |
| TOPCONS | iiiiiiiiii iiiiiiiiii iiiiiiiiii iiiiiiiiii iiiMMMMMMM |
| OCTOPUS | iiiiiiiiii iiiiiiiiii iiiiiiiiii iiiiiiiiii iiiMMMMMMM |
| Philius | iiiiiiiiii iiiiiiiiii iiiiiiiiii iiiiiiiiii iiiiiiMMMM |
| PolyPhobius | iiiiiiiiii iiiiiiiiii iiiiiiiiii iiiiiiiiii iiiiiiMMMM |
| SCAMPI | iiiiiiiiii iiiiiiiiii iiiiiiiiii iiiiiiiiii iiiMMMMMMM |
| SPOCTOPUS | iiiiiiiiii iiiiiiiiii iiiiiiiiii iiiiiiiiii iiiMMMMMMM |
| PDB-homology |  |
|  | |
|  | 501                                         541 |
| Seq. | TFILIFFESF GTLYYILNAI MDMDRKEADH YNFTGVVVRL ELSSSALLHM |
| TOPCONS | MMMMMMMMMM MMMMoooooo oooooooooo oooooooooo MMMMMMMMMM |
| OCTOPUS | MMMMMMMMMM MMMMoooooo oooooooooo oooooooMMM MMMMMMMMMM |
| Philius | MMMMMMMMMM MMMMMMMooo oooooooooo oooooooooo oMMMMMMMMM |
| PolyPhobius | MMMMMMMMMM MMMMMMMooo oooooooooo oooooooooo oMMMMMMMMM |
| SCAMPI | MMMMMMMMMM MMMMoooooo oooooooooo oooooooooo MMMMMMMMMM |
| SPOCTOPUS | MMMMMMMMMM MMMMoooooo oooooooooo oooooooMMM MMMMMMMMMM |
| PDB-homology |  |
|  | |
|  | 551                                         591 |
| Seq. | MGLIFNVRAI LIMTHRLRAV HTIASEQHAH LTCKMDLMKD EDDEKEMLKG |
| TOPCONS | MMMMMMMMMM Miiiiiiiii iiiiiiiiii iiiiiiiiii iiiiiiiiii |
| OCTOPUS | MMMMMMMMii iiiiiiiiii iiiiiiiiii iiiiiiiiii iiiiiiiiii |
| Philius | MMMMMMMMMM MMiiiiiiii iiiiiiiiii iiiiiiiiii iiiiiiiiii |
| PolyPhobius | MMMMMMMMMM Miiiiiiiii iiiiiiiiii iiiiiiiiii iiiiiiiiii |
| SCAMPI | MMMMMMMMMM Miiiiiiiii iiiiiiiiii iiiiiiiiii iiiiiiiiii |
| SPOCTOPUS | MMMMMMMMii iiiiiiiiii iiiiiiiiii iiiiiiiiii iiiiiiiiii |
| PDB-homology |  |
|  | |
|  | 601                                         641 |
| Seq. | LGLEFEQYMK RQSLLQYMVN HPLGITIYGF LIDREFLRSF HMVIVSLTVF |
| TOPCONS | iiiiiiiiii iiiiiiiiii iiiiiiiiii iiiiiiMMMM MMMMMMMMMM |
| OCTOPUS | iiiiiiiiii iiiiiiiiii iiiiiiiiii iiiiiMMMMM MMMMMMMMMM |
| Philius | iiiiiiiiii iiiiiiiiii iiiiiiiiii iiiiiiiiMM MMMMMMMMMM |
| PolyPhobius | iiiiiiiiii iiiMMMMMMM MMMMMMMMMM MMoooooMMM MMMMMMMMMM |
| SCAMPI | iiiiiiiiii iiiiiiiiii iiiiiiiiii iiiiiMMMMM MMMMMMMMMM |
| SPOCTOPUS | iiiiiiiiii iiiiiiiiii iiiiiiiiii iiiiiiMMMM MMMMMMMMMM |
| PDB-homology |  |
|  | |

|  |  |
| --- | --- |
|  | 651        661 |
| Seq. | LVSMIMGGSH SSQSGGNHKA |
| TOPCONS | MMMMMMMooo oooooooooo |
| OCTOPUS | MMMMMMoooo oooooooooo |
| Philius | MMMMMMMMMM oooooooooo |
| PolyPhobius | MMMMMMMMii iiiiiiiiii |
| SCAMPI | MMMMMMoooo oooooooooo |
| SPOCTOPUS | MMMMMMMooo oooooooooo |
| PDB-homology |  |
