# Supplementary material for: A putative origin of the insect chemosensory receptor superfamily in the last common eukaryotic ancestor
Source: eLife. 2020 Dec 4;9:e62507. doi: 10.7554/eLife.62507 (PMC7746228; doi:10.7554/eLife.62507)
Supplement: Supplementary file 2. [file elife-62507-supp2.zip › 201130_SuppFile2_TOPCONS/seq_12/nicetop.html]

|  |  |
| --- | --- |
|  | 1                                           41 |
| Seq. | MTLASVEREK QDDTADRPPL GSVPCGRECQ SDALRQSAAI HIASLSLYAD |
| TOPCONS | iiiiiiiiii iiiiiiiiii iiiiiiiiii iiiiiiiiii iiiiiiiiii |
| OCTOPUS | iiiiiiiiii iiiiiiiiii iiiiiiiiii iiiiiiiiii iiiiiiiiii |
| Philius | iiiiiiiiii iiiiiiiiii iiiiiiiiii iiiiiiiiii iiiiiiiiii |
| PolyPhobius | iiiiiiiiii iiiiiiiiii iiiiiiiiii iiiiiiiiii iiiiiiiiii |
| SCAMPI | iiiiiiiiii iiiiiiiiii iiiiiiiiii iiiiiiiiii iiiiiiiiii |
| SPOCTOPUS | iiiiiiiiii iiiiiiiiii iiiiiiiiii iiiiiiiiii iiiiiiiiii |
| PDB-homology |  |
|  | |
|  | 51                                          91 |
| Seq. | LQHTNGGIDQ HTWHRAKQAL DAFRNDMQLP GTSASDHDDA GRRGTQQHRQ |
| TOPCONS | iiiiiiiiii iiiiiiiiii iiiiiiiiii iiiiiiiiii iiiiiiiiii |
| OCTOPUS | iiiiiiiiii iiiiiiiiii iiiiiiiiii iiiiiiiiii iiiiiiiiii |
| Philius | iiiiiiiiii iiiiiiiiii iiiiiiiiii iiiiiiiiii iiiiiiiiii |
| PolyPhobius | iiiiiiiiii iiiiiiiiii iiiiiiiiii iiiiiiiiii iiiiiiiiii |
| SCAMPI | iiiiiiiiii iiiiiiiiii iiiiiiiiii iiiiiiiiii iiiiiiiiii |
| SPOCTOPUS | iiiiiiiiii iiiiiiiiii iiiiiiiiii iiiiiiiiii iiiiiiiiii |
| PDB-homology |  |
|  | |
|  | 101                                         141 |
| Seq. | QGRQAISTSS CEPMPEPHES ELRVRGSAST RAPSPNYEAP LCVAATSTML |
| TOPCONS | iiiiiiiiii iiiiiiiiii iiiiiiiiii iiiiiiiiii iiiiiiiiii |
| OCTOPUS | iiiiiiiiii iiiiiiiiii iiiiiiiiii iiiiiiiiii iiiiiiiiii |
| Philius | iiiiiiiiii iiiiiiiiii iiiiiiiiii iiiiiiiiii iiiiiiiiii |
| PolyPhobius | iiiiiiiiii iiiiiiiiii iiiiiiiiii iiiiiiiiii iiiiiiiiii |
| SCAMPI | iiiiiiiiii iiiiiiiiii iiiiiiiiii iiiiiiiiii iiiiiiiiii |
| SPOCTOPUS | iiiiiiiiii iiiiiiiiii iiiiiiiiii iiiiiiiiii iiiiiiiiii |
| PDB-homology |  |
|  | |
|  | 151                                         191 |
| Seq. | KPEISQVSSR DSVGWLQWCA MIFMHPKHYE CHAPELDETV QFMHQGTPLE |
| TOPCONS | iiiiiiiiii iiiiiiiiii iiiiiiiiii iiiiiiiiii iiiiiiiiii |
| OCTOPUS | iiiiiiiiii iiiiiiiiii iiiiiiiiii iiiiiiiiii iiiiiiiiii |
| Philius | iiiiiiiiii iiiiiiiiii iiiiiiiiii iiiiiiiiii iiiiiiiiii |
| PolyPhobius | iiiiiiiiii iiiiiiiiii iiiiiiiiii iiiiiiiiii iiiiiiiiii |
| SCAMPI | iiiiiiiiii iiiiiiiiii iiiiiiiiii iiiiiiiiii iiiiiiiiii |
| SPOCTOPUS | iiiiiiiiii iiiiiiiiii iiiiiiiiii iiiiiiiiii iiiiiiiiii |
| PDB-homology |  |
|  | |
|  | 201                                         241 |
| Seq. | TWWVRVAGLS PSSSDSRLVR QIWPIIIHIV LWYGAICNGI IFFYSAVDPE |
| TOPCONS | iiiiiiiiii iiiiiiiiiM MMMMMMMMMM MMMMMMMMMM oooooooooo |
| OCTOPUS | iiiiiiiiii iiiiiiiiiM MMMMMMMMMM MMMMMMMMMM oooooooooo |
| Philius | iiiiiiiiii iiiiiiiiii iiiiMMMMMM MMMMMMMMMM MMMMMMoooo |
| PolyPhobius | iiiiiiiiii iiiiiiiiii iiiMMMMMMM MMMMMMMMMM MMMMMMMooo |
| SCAMPI | iiiiiiiiii iiiiiiMMMM MMMMMMMMMM MMMMMMMooo oooooooooo |
| SPOCTOPUS | iiiiiiiiii iiiiiiiiiM MMMMMMMMMM MMMMMMMMMM oooooooooo |
| PDB-homology |  |
|  | |
|  | 251                                         291 |
| Seq. | WCDRSYSPEW CGGREDLLLL FMFWSIALAV AVAYTVLRVQ WICRGNAFLY |
| TOPCONS | oooooooooo ooooooMMMM MMMMMMMMMM MMMMMMMiii iiiiiiiiii |
| OCTOPUS | oooooooooo ooooooMMMM MMMMMMMMMM MMMMMMMiii iiiiiiiiii |
| Philius | oooooooooo ooooooMMMM MMMMMMMMMM MMMMMMMiii iiiiiiiiii |
| PolyPhobius | oooooooooo ooooooMMMM MMMMMMMMMM MMMMMMMiii iiiiiiiiii |
| SCAMPI | oooooooooo ooooooMMMM MMMMMMMMMM MMMMMMMiii iiiiiiiiii |
| SPOCTOPUS | oooooooooo oooooooMMM MMMMMMMMMM MMMMMMMMii iiiiiiiiii |
| PDB-homology |  |
|  | |
|  | 301                                         341 |
| Seq. | ALDFIRRECK DEKNLLNRLT RDVRSKFTWT MVFTLLFWGY FFYIGVIYRV |
| TOPCONS | iiiiiiiiii iiiiiiiiii iiiiiiMMMM MMMMMMMMMM MMMMMMMooo |
| OCTOPUS | iiiiiiiiii iiiiiiiiii iiiiiMMMMM MMMMMMMMMM MMMMMMoooo |
| Philius | iiiiiiiiii iiiiiiiiii iiiiiiMMMM MMMMMMMMMM MMMMMMMooo |
| PolyPhobius | iiiiiiiiii iiiiiiiiii iiiiiiMMMM MMMMMMMMMM MMMMMMMMoo |
| SCAMPI | iiiiiiiiii iiiiiiiiii iiiiiiMMMM MMMMMMMMMM MMMMMMMooo |
| SPOCTOPUS | iiiiiiiiii iiiiiiiiii iiiiiMMMMM MMMMMMMMMM MMMMMMoooo |
| PDB-homology |  |
|  | |
|  | 351                                         391 |
| Seq. | AFATDRQKKY WGAFPGLHTW PIFREINSYV LDVASTLFEP FIGMTVAAQT |
| TOPCONS | oooooooooo oooooooooo oooooooooo oooooooooo MMMMMMMMMM |
| OCTOPUS | oooooooooo oooooooooo oooooooooo oMMMMMMMMM MMMMMMMMMM |
| Philius | oooooooooo oooooooooo oooooooooo oooooooooo MMMMMMMMMM |
| PolyPhobius | oooooooooo oooooooooo oooooooooo oooooooooo MMMMMMMMMM |
| SCAMPI | oooooooooo oooooooooo oooooooooo oooooooooo MMMMMMMMMM |
| SPOCTOPUS | oooooooooo oooooooooo oooooooooo oMMMMMMMMM MMMMMMMMMM |
| PDB-homology |  |
|  | |
|  | 401                                         441 |
| Seq. | GIICTIHRSS FNVLMYHMLK RDRRIHRNGT TGDDDVDTPL SVPQLIEMHR |
| TOPCONS | MMMMMMMMMM Miiiiiiiii iiiiiiiiii iiiiiiiiii iiiiiiiiii |
| OCTOPUS | MMiiiiiiii iiiiiiiiii iiiiiiiiii iiiiiiiiii iiiiiiiiii |
| Philius | MMMMMMMMMM Miiiiiiiii iiiiiiiiii iiiiiiiiii iiiiiiiiii |
| PolyPhobius | MMMMMMMMMM MMMMMMiiii iiiiiiiiii iiiiiiiiii iiiiiiiiii |
| SCAMPI | MMMMMMMMMM Miiiiiiiii iiiiiiiiii iiiiiiiiii iiiiiiiiii |
| SPOCTOPUS | MMiiiiiiii iiiiiiiiii iiiiiiiiii iiiiiiiiii iiiiiiiiii |
| PDB-homology |  |
|  | |
|  | 451                                         491 |
| Seq. | FLDGMLNKSS HILELPITAM GTLYCISFLS CLYLLLFRPP VGETGQLDLF |
| TOPCONS | iiiiiiiiii iiiMMMMMMM MMMMMMMMMM MMMMoooooo oooooooMMM |
| OCTOPUS | iiiiiiiiii iiiMMMMMMM MMMMMMMMMM MMMMoooooo ooooooMMMM |
| Philius | iiiiiiiiii iiiiMMMMMM MMMMMMMMMM MMMMMMMooo ooooooooMM |
| PolyPhobius | iiiiiiiiii iiiMMMMMMM MMMMMMMMMM MMMMMMMooo ooooooooMM |
| SCAMPI | iiiiiiiiii iMMMMMMMMM MMMMMMMMMM MMoooooooo oooooooMMM |
| SPOCTOPUS | iiiiiiiiii iiiMMMMMMM MMMMMMMMMM MMMMoooooo ooooooMMMM |
| PDB-homology |  |
|  | |
|  | 501                                         541 |
| Seq. | FVIVAIFFCI GVCMSMILSD TAKVTAKCAR LAEIASFASR HYQLYGEHPP |
| TOPCONS | MMMMMMMMMM MMMMMMMMii iiiiiiiiii iiiiiiiiii iiiiiiiiii |
| OCTOPUS | MMMMMMMMMM MMMMMMMiii iiiiiiiiii iiiiiiiiii iiiiiiiiii |
| Philius | MMMMMMMMMM MMMMMMMMii iiiiiiiiii iiiiiiiiii iiiiiiiiii |
| PolyPhobius | MMMMMMMMMM MMMMMMMMii iiiiiiiiii iiiiiiiiii iiiiiiiiii |
| SCAMPI | MMMMMMMMMM MMMMMMMMii iiiiiiiiii iiiiiiiiii iiiiiiiiii |
| SPOCTOPUS | MMMMMMMMMM MMMMMMMiii iiiiiiiiii iiiiiiiiii iiiiiiiiii |
| PDB-homology |  |
|  | |
|  | 551                                         591 |
| Seq. | PNSPQYARTR LQGLVSDRTM SRAVAESISK GACSATTREV TTDKNKRPDI |
| TOPCONS | iiiiiiiiii iiiiiiiiii iiiiiiiiii iiiiiiiiii iiiiiiiiii |
| OCTOPUS | iiiiiiiiii iiiiiiiiii iiiiiiiiii iiiiiiiiii iiiiiiiiii |
| Philius | iiiiiiiiii iiiiiiiiii iiiiiiiiii iiiiiiiiii iiiiiiiiii |
| PolyPhobius | iiiiiiiiii iiiiiiiiii iiiiiiiiii iiiiiiiiii iiiiiiiiii |
| SCAMPI | iiiiiiiiii iiiiiiiiii iiiiiiiiii iiiiiiiiii iiiiiiiiii |
| SPOCTOPUS | iiiiiiiiii iiiiiiiiii iiiiiiiiii iiiiiiiiii iiiiiiiiii |
| PDB-homology |  |
|  | |
|  | 601                                         641 |
| Seq. | GRFTRRLLRV WRRVKKEHSE RREGDLEAGL DREDTLGSRV HLVEEVGAPV |
| TOPCONS | iiiiiiiiii iiiiiiiiii iiiiiiiiii iiiiiiiiii iiiiiiiiii |
| OCTOPUS | iiiiiiiiii iiiiiiiiii iiiiiiiiii iiiiiiiiii iiiiiiiiii |
| Philius | iiiiiiiiii iiiiiiiiii iiiiiiiiii iiiiiiiiii iiiiiiiiii |
| PolyPhobius | iiiiiiiiii iiiiiiiiii iiiiiiiiii iiiiiiiiii iiiiiiiiii |
| SCAMPI | iiiiiiiiii iiiiiiiiii iiiiiiiiii iiiiiiiiii iiiiiiiiii |
| SPOCTOPUS | iiiiiiiiii iiiiiiiiii iiiiiiiiii iiiiiiiiii iiiiiiiiii |
| PDB-homology |  |
|  | |
|  | 651                                         691 |
| Seq. | QKARTQADTP TGNLSEAMQQ ILLVQYLQAS NTSWRVYGVK MTSTVQGRIL |
| TOPCONS | iiiiiiiiii iiiiiiiiii iiiiiiiiii iiiiiiiiii iiiiiiiMMM |
| OCTOPUS | iiiiiiiiii iiiiiiiiii iiiiiiiiii iiiiiiiiii iiiiiiiiii |
| Philius | iiiiiiiiii iiiiiiiiii iiiiiiiiii iiiiiiiiii iiiiiiiiMM |
| PolyPhobius | iiiiiiiiii iiiiiiiiii iiiiiiiiii iiiiiiiiii iiiiiiiMMM |
| SCAMPI | iiiiiiiiii iiiiiiiiii iiiiiiiiii iiiiiiiiii iiiiMMMMMM |
| SPOCTOPUS | iiiiiiiiii iiiiiiiiii iiiiiiiiii iiiiiiiiii iiiiiiiiii |
| PDB-homology |  |
|  | |

|  |  |
| --- | --- |
|  | 701        711 |
| Seq. | YTVGTLIAVG LQRALSTSFS |
| TOPCONS | MMMMMMMMMM MMMMMMMMoo |
| OCTOPUS | iiiiiiiiii iiiiiiiiii |
| Philius | MMMMMMMMMM MMMMMooooo |
| PolyPhobius | MMMMMMMMMM MMMMMooooo |
| SCAMPI | MMMMMMMMMM MMMMMooooo |
| SPOCTOPUS | iiiiiiiiii iiiiiiiiii |
| PDB-homology |  |
